# Clinical and Economic Outcomes of Transcutaneous Afferent Patterned Stimulation for Essential Tremor

**DOI:** 10.36469/001c.170793

**Published:** 2026-10-22

**Authors:** Dingwei Dai, Chiahao Lu, Kathryn H. Rosenbluth, Joaquim Fernandes

**Affiliations:** 1 CVS Healthspire™ Life Sciences Solution, Woonsocket, Rhode Island, USA; 2 Cala Health, Inc., San Mateo, California, USA

**Keywords:** essential tremor, transcutaneous afferent patterned stimulation, pragmatic clinical trial, external control arm, activities of daily living, healthcare resource utilization, healthcare costs

## Abstract

**Background:**

TAPS® therapy (Cala Health) delivers transcutaneous afferent patterned stimulation through a noninvasive, wrist-worn neuromodulation device for the treatment of hand tremor in patients with essential tremor (ET). Although previous studies have demonstrated its effectiveness and safety, real-world evidence regarding its long-term clinical outcomes and economic impact remains limited.

**Objectives:**

To evaluate the long-term clinical effectiveness and the healthcare resource utilization and cost impact of TAPS in patients with ET treated in real-world settings.

**Methods:**

Patients who completed a 1-month randomized pragmatic phase comparing TAPS with standard of care entered 12 months of open-label home use. Clinical outcomes were assessed via device-based motion sensors and the Bain & Findley Activities of Daily Living (BF-ADL) upper-limb scale at baseline and every 3 months. Healthcare resource utilization and costs over the 12-month TAPS treatment period were compared with those during the 12 months before TAPS initiation and with a propensity-score-matched external control arm (ECA) derived from a large US commercial and Medicare Advantage claims database.

**Results:**

Among 225 patients who used TAPS therapy for 12 months, daily functioning improved, with the mean BF-ADL score decreasing from 17.2 to 15.8 (*P* < .003). Device-measured tremor power decreased by more than 50% (*P* < .001). No serious device-related adverse events were reported. In the ECA analysis, 153 TAPS-treated patients were matched to 306 ECA patients. Compared with baseline, TAPS-treated patients experienced significant reductions in ET-related healthcare costs ($440 decrease; *P* < .0001), ET-related medication use (15% fewer patients; *P* < .01), and ambulatory and office visits (~0.3 fewer each; *P* < .05). These reductions were significantly greater than those observed in the ECA (all *P* < .05).

**Discussion:**

This is the largest and longest prospective ET study to jointly assess tremor severity, daily function, and healthcare economic outcomes. The sustained clinical benefits observed with TAPS therapy, together with reductions in ET-related healthcare utilization and costs, support TAPS as a noninvasive treatment option for adults with ET.

**Conclusions:**

Over 12 months of real-world use, TAPS reduced tremor severity, improved daily functioning, and lowered ET-related healthcare resource utilization and costs.

## INTRODUCTION

Essential tremor (ET) is one of the most common neurodegenerative movement disorders in adults and affects approximately 7 million individuals in the United States (US) and 25 million globally.^1,2^ A large US administrative claims study reported a consistent annual increase in prevalence from 2010 to 2018, particularly among Medicare Advantage beneficiaries.^3^ These findings highlight ET’s growing impact on aging populations. Clinically, most patients exhibit a postural and kinetic tremor in the upper limbs. While early symptoms are often mild, 30% to 50% of individuals eventually experience progression to more severe symptoms, posting considerable clinical challenges.^1,4–6^ Increased tremor severity interferes with activities of daily living (ADL) such as eating, drinking, writing, and self-care, contributing to functional impairment, social embarrassment, and decreased quality of life.^6–9^

The chronic and progressive nature of ET requires long-term management strategies, which place a substantial burden on healthcare systems globally. While first-line medications including propranolol and primidone are widely used and inexpensive,^6,7,10^ the true management burden stems from medication intolerance and lack of efficacy. This frequently leads to medication discontinuation for most patients.^11,12^ Up to 55% of patients are considered refractory to these pharmacological treatments.^13^ Although surgical options like deep brain stimulation (DBS), radio-frequency thalamotomy and magnetic resonance-guided focused ultrasound (MRgFUS) are effective, they are costly and carry substantial risks, including intracranial hemorrhage, paresthesia, numbness, and gait disturbances.^7,12,14–16^ This treatment gap may partially explain the increased healthcare resource utilization (HCRU) and costs reported in recent epidemiological and real-world evidence studies.^4,7–9,17^

An alternative treatment, Transcutaneous Afferent Patterned Stimulation therapy (TAPS® therapy, Cala Health, Inc.) delivers transcutaneous afferent patterned stimulation via a noninvasive, individually calibrated neuromodulation device to nerves at the wrist to reduce postural and kinetic tremors in patients with ET or Parkinson’s disease. TAPS devices (Cala Health) measure each patient’s unique tremor signature and deliver an alternating stimulation pulse train to the median and radial peripheral nerves that is calibrated to each patient’s tremor frequency.^18–23^ TAPS can be used adjunctively to pharmacological treatments or independently.^23–25^ Previous studies have shown that each use of TAPS provides sustained tremor reduction for at least 1 hour after therapy and can reduce underlying tremor when used daily.^20,21^

This study reports the 12-month outcomes of a large prospective, randomized pragmatic study on TAPS (ClinicalTrials.gov: NCT05540626). The 1-month results of the study included a comparison between TAPS and standard of care (SOC) and have been previously published, showing that adding TAPS to SOC significantly improved tremor severity (primary endpoint) and ADLs (secondary endpoint) during the initial 1-month period compared with SOC alone.^24^ While these findings established the short-term effectiveness of TAPS, understanding the long-term clinical durability and real-world economic impact is critical for evaluating its role in chronic ET management. Therefore, this study reports on the subsequent phase, in which all patients crossed over to 12 months of open-label home use, to prospectively evaluate whether TAPS reduces tremor severity, improve ADLs, and decreases ET-related HCRU and associated costs. These outcomes are increasingly important to payers and coverage decision makers evaluating noninvasive treatment alternatives.

## METHODS

### Study Design

We conducted a prospective, 12-month open-label extension of a randomized pragmatic trial to assess the clinical effectiveness of TAPS therapy, with an external claims-based control arm to compare HCRU and costs between patients with ET receiving TAPS therapy and those receiving non-TAPS routine clinical care. As previously reported, patients with ET were identified in the claims database of a major payor (CVS Aetna) in the US and electronically consented between May 2021 and June 2023.^24^ Patients were eligible if they had at least 2 medical claims with an ET diagnosis code (*International Classification of Disease, 10th Revision, Clinical Modification* (ICD-10-CM): G25.0), were at least 22 years of age, and had continuous medical and pharmacy health insurance coverage for at least 12 months prior to enrollment. Additional inclusion and exclusion criteria have been previously described.^24^

As previously reported, patients were randomized to either a treatment (TX) or SOC arm for the first month of the study, Patients in the TX arm added TAPS therapy to their physician-recommended care plan while patients in the SOC arm continued with their existing care plan.^24^ Patients were instructed to self-administer TAPS as needed to relieve tremor symptoms, such as ADLs requiring hand control, including eating, drinking, or writing.

After the 1-month randomized period, patients in the SOC crossed over into TX, and all patients continued with TAPS therapy and their physician-recommended care for 12 months. Patients crossing over from SOC to TAPS received the same instructions that patients randomized to TX received at the start of the study, including instructions on performing postural holds before and after stimulation sessions for measuring tremor power, self-administering 40-minute stimulation sessions to control their tremor, reporting adverse events or device issues, a reminder to continue with any other ET-related care and comorbidity management recommended by their physician, and instructions for completing the Bain & Findley ADL (BF-ADL) upper limb rating scale at 3-, 6-, 9-, and 12-month follow-up periods. Importantly, the study protocol did not require patients to maintain a fixed medication dose; instead, dose adjustments or discontinuations were permitted at the treating physician’s discretion to reflect standard clinical practice.

For the health economic analyses, patients with ET who enrolled in the clinical trial and received 12 months of TAPS therapy were matched in a 1:2 ratio to external patients with ET not receiving TAPS therapy, identified from the payor’s claims database, using propensity scores (external control arm, ECA). The non-TAPS ET patients were selected from the payor’s claims database who met the TAPS study eligibility criteria (**Supplementary Figure S1**).^24^ The index date was defined as the first date of TAPS therapy for patients in the TAPS arm. For the ECA, the index date was randomly assigned after each patient’s initial ET diagnosis claim, ensuring that the assigned date fell within the same calendar period during which TAPS patients initiated TAPS therapy. The 12 months prior to the index date were defined as the baseline period, and the 12 months following the index date were defined as the follow-up period. Matching factors included age, gender, geographic region, household income, social vulnerability index, payer type, top 25 comorbidities, Charlson Comorbidity Index (CCI), ET treatments, healthcare costs, and index year. Balance between the two arms across all potential confounders was assessed using standardized mean differences. The full details of propensity score matching are provided in the **Supplementary Methods**.

The study was approved by the Sterling Institutional Review Board (Atlanta, Georgia, USA; IRB ID: 8725-HCoetzer) on March 3, 2021, prior to implementation of any study procedures, and was registered with ClinicalTrials.gov (NCT05540626). Electronic informed consent was obtained from all study participants who received TAPS therapy. This study followed the Consolidated Standards of Reporting Trials (CONSORT) and the Strengthening the Reporting of Observational Studies in Epidemiology (STROBE) guidelines.^26,27^

### Data Collection

Demographic and clinical data were collected either directly from patients or derived from the CVS Aetna health claims database.

Device usage, tremor motion data and patient-reported change in tremor severity were recorded by the device and uploaded remotely via a cloud-based system to the manufacturer’s secured database, which contained timestamps of all sessions and the accelerometer measurements during postural holds prompted by the device.^20,23,24^ The device prompted patients to perform a postural hold for collecting motion data immediately before and after each session, and to select one of three options for their change in tremor severity (ie, improved, no change, or worsened) after each session.

BF-ADL upper limb scores were collected using patient surveys administered on the e-consent platform at baseline and at follow-up periods: 3, 6, 9 and 12 months.^24,28^

HCRU and cost data were extracted from the Aetna claims database, which has been described previously.^4,24^

To assess comorbid conditions, claims data were analyzed using previously described methods based on ICD-10-CM codes.^4,10^ The CCI was then calculated based on the claims data.^4,29^

### Clinical Outcome Measures

The 1-month change in median tremor power between the TX and SOC arms served as the primary clinical endpoint and has been previously reported.^24^ Here, we presented the planned longitudinal analyses of tremor power reduction and BF-ADL improvement at 1-3, 4-6, 7-9, 10-12 months, and over 1-12 months.

Tremor power reduction was measured during postural holds from pre- to post-stimulation during the prespecified follow-up periods. Tremor power is a metric capturing the frequency and amplitude of tremor motion and has been previously shown to correlate with the Tremor Research Group Essential Tremor Rating Assessment Scale (TETRAS).^20,21,30^ It was computed from the power spectral density of the postural hold device accelerometry data. Specifically, the spectral peak was identified within the 4 to 12 Hz range expected for ET, after which tremor power was calculated by integrating the power spectral density over a 3 Hz window centered on the identified spectral peak.^22–24^ The median tremor power improvement ratio (TPIR) was determined for each patient across TAPS sessions during the follow-up periods, representing the ratio of the pre- to post-stimulation tremor power.^20^ A TPIR of 1 indicated no change, a ratio greater than 1 signified tremor improvement (eg, a 2-fold improvement ratio corresponding to a 50% tremor reduction) and a ratio less than 1 indicated tremor worsening. Finally, the percentage of sessions that a patient self-rated as tremor improved was reported.

Improvement in BF-ADL upper limb score was evaluated at the prespecified follow-up periods (3, 6, 9, and 12 months) using responses from the patient reported surveys.

### Health Economic Outcome Measures

The prespecified health economic outcomes were the change in HCRU and costs from the 12 months prior to enrollment to the 12 months of study enrollment, as compared between the enrolled patients and the ECA. HCRU and costs were analyzed, including ET-related medications (propranolol, primidone, topiramate, gabapentin, alprazolam, clonazepam, diazepam, metoprolol, and atenolol) and other medications (antidepressants, anxiolytics, and opioids), ambulatory visits (eg, public health clinics, urgent care, therapeutic services, outpatient surgery/procedure centers, rehabilitation services, diagnostic imaging [MRI, x-ray], laboratory tests, telehealth, and nurse home visits, excluding specialist and primary care physician [PCP] visits), office visits with PCPs, specialist visits (eg, neurologist, cardiologist, psychiatrist), inpatient admission, and emergency department (ED) visits. Healthcare costs were categorized as medical, pharmacy, and total costs. ET-related HCRU and costs were extracted from medical claims with an ICD-10-CM code for ET (G25.0) in any position and pharmacy claims for an ET treatment.^4,24,31^ All costs were inflated to 2023 US dollars using the medical care services component of the Consumer Price Index.^32^

Health economic outcomes, including HCRU and costs, were assessed by comparing values from 12 months prior to the index date (12-month baseline) to 12 months following the index date (12-month follow-up). Comparisons were made within each group (baseline vs follow-up) and between groups (TAPS vs ECA) based on changes across these two time periods.

### Severe ET Subgroup Analyses

A subgroup of more severe patients was defined as having at least 1 eating, drinking, or writing task with a BF-ADL score of 3 or 4 at baseline.^22,24^ The clinical and health economic outcome measures were analyzed within this subgroup.

### Statistical Analysis

Categorical variables were described as the number of observations and percentage. Continuous variables were described as means with standard deviations (SD) or medians with interquartile ranges (IQR), as appropriate. Tremor power and TPIR were extracted as the median for each patient and then reported as geometric mean and geometric standard error, equivalent in range to the mean and standard error of log-transformed data, as tremor power is logarithmically distributed. Improvement in BF-ADL scores were reported using mean and SD.

Differences in clinical outcome measures between the pre-TAPS therapy baseline and the specific post-TAPS therapy follow-up periods were tested using a paired *t*-test or the Wilcoxon signed-rank test for continuous variables and McNemar or McNemar-Bowker test for categorical variables.

To evaluate changes in tremor power improvement over time, a linear mixed effects model was used with median TPIR as the dependent variable, follow-up periods (months 1-3, 4-6, 7-9, 10-12) as the fixed factor, and patient as the random-intercept term. To examine whether pre- and post-stimulation tremor severity changed during the 12-month follow-up, a similar linear mixed effects model was used, with median tremor power (pre- or post-stimulation) as the dependent variable. TPIR and tremor power were log-transformed before entering the mixed model or *t*-test to account for skewness.^23,30^ Changes in ADLs were assessed using another linear mixed effects model with BF-ADL as the dependent variable, patient as the random-intercept term, and follow-up timepoints (months 3, 6, 9, 12) as the fixed factor. Significance was tested using *F*-tests with Satterthwaite’s approximation for degrees of freedom and Tukey adjustment for pairwise comparisons. Changes in BF-ADL severity category (moderate/severe to mild/no tremor) between baseline and each follow-up were evaluated using the chi-square test.

HCRU and costs were compared between TAPS patients and the ECA. Negative binomial regression with a log-link function was performed to evaluate the association between TAPS therapy and HCRU, including ambulatory, office, specialist (neurologist, cardiologist, psychiatrist, and other), nurse practitioner and ED visits, inpatient admissions, and ET-related medication use. Generalized linear models were performed to evaluate the association between TAPS therapy and healthcare costs. A gamma distribution with a log-link function was used to account for the right-skewed distribution and heteroskedasticity commonly observed in healthcare cost data.^33^ If all covariables were balanced (ie, standardized mean difference, <0.1) after propensity score matching (PSM), the univariate generalized linear models which contained exposure status (TAPS therapy) as the sole predictor were performed. Otherwise, multivariable generalized linear models were fit to the matched cohort and included the exposure status and remaining unbalanced covariables.

Statistical analyses were conducted using SAS version 9.4 (SAS Institute Inc.). For longitudinal clinical outcomes, Holm-Bonferroni corrections were applied to the family of pairwise comparisons between baseline and each follow-up time point within each outcome measure (TPIR and BF-ADL scores) to control the family-wise error rate. Missing data were reported as observed and were not replaced or otherwise imputed. Analyses were conducted using available data for each outcome measure. All *P* values were two-sided, with *P* < .05 considered statistically significant. Adverse events (AEs), severe ET subgroup analysis, and TAPS therapy device usage analyses were performed as described previously (see **Supplementary Material** for other subgroup analyses, eg, age ≥65 vs age <65).^24^ To evaluate the robustness of the economic findings across key demographics and clinical subgroups, supplementary subgroup analyses of healthcare costs were conducted comparing the TAPS and ECA cohorts specifically among patients aged ≥65 years and those with prior use of ET-related medications at baseline.

## RESULTS

### Baseline Characteristics

The study enrolled 310 patients, of which 276 patients completed the 1-month randomized phase.^24^ A total of 225 patients completed the study by continuing to use the device for 12 months, whereas 51 patients (18%) withdrew between months 1 and 12 for various reasons (**Supplementary Figure S2, Table S1** for reasons for withdrawal). The most common reasons for withdrawal included lack of benefit (N = 11, 22% of withdrawals) and no longer wishes to participate (N = 10, 20% of withdrawals). Among the 225 patients who completed the study, the number of patients completing assessments varied by outcome measure. For example, 148 patients completed the 12-month BF-ADL survey.

**Supplementary Table S2** compares baseline characteristics between patients who completed and those who discontinued the 12-month pragmatic clinical trial. Baseline demographics, comorbidity burden, ET-related medication use, BF-ADL scores, and tremor power were generally similar between groups (all *P* > .05). The only significant difference was insurance type, with discontinuers more likely to have Medicare Advantage coverage (*P* = .0021), suggesting limited attrition bias.

**Table 1** presents baseline characteristics for the 225 patients who completed the study. The mean age was 67.7 years, with 72.4% aged 65 or older, 67.6% male, and 70% had received ET-related pharmacotherapy within 12 months prior to initiating TAPS therapy (including 32% with propranolol and 29% with primidone). On average, patients had 7.3 comorbid conditions.

**Table 1.** Baseline Clinical Characteristics

**Characteristics**	**Study Patients (N = 225)**
Age (years), mean (SD)	67.7 (11.1)
Age group, % (N)	
22-44 years	4.0 (9)
45-64 years	23.6 (53)
65-74 years	44.9 (101)
≥75 years	27.6 (62)
Gender, % (N)	
Male	67.6 (152)
Comorbidity	
Charlson Comorbidity Index, mean (SD)	2.12 (2.27)
No. of comorbidities, mean (SD)	7.3 (3.0)
Top 10 most common comorbidities % (N)	
Pain disorders	84.4 (190)
Hyperlipidemia	81.8 (184)
Hypertension	68.4 (154)
Fatigue and sleep-related disorders	44.0 (99)
Obesity	41.8 (94)
Osteoarthritis	41.3 (93)
Anxiety	30.2 (68)
Chronic pulmonary disease	29.3 (66)
Diabetes mellitus	25.8 (58)
Depression	22.7 (51)
Medication use, % (N)	
ET-related medications	
Propranolol	32.0 (72)
Primidone	29.3 (66)
Topiramate	8.0 (18)
Gabapentin	15.6 (35)
Alprazolam	4.9 (11)
Clonazepam	4.4 (10)
Diazepam	1.3 (3)
Metoprolol	15.1 (34)
Atenolol	2.7 (6)
Any medications	69.8 (157)
Other medications	
Antidepressants	26.2 (59)
Anxiolytics	14.7 (33)
Opioid use	18.2 (41)
Baseline BF-ADL severity^a^, % (N)	
BF-ADL severity ≥2	95.1 (214)
BF-ADL severity ≥3	82.7 (186)
BF-ADL severity = 4	28.0 (63)

Abbreviations: BF-ADL, Bain and Findley Activities of Daily Living Scale; CCI, Charlson Comorbidity Index; ET, essential tremor.

^a^BF-ADL tasks associated with eating, drinking, or writing (ie, use a spoon to drink soup; hold a cup of tea; pour milk from a bottle; write a letter). Patients were classified as having at least 1 of these 4 BF-ADL tasks with a score of ≥2, ≥3, or 4.

### Clinical Outcomes

**Figure 1.**
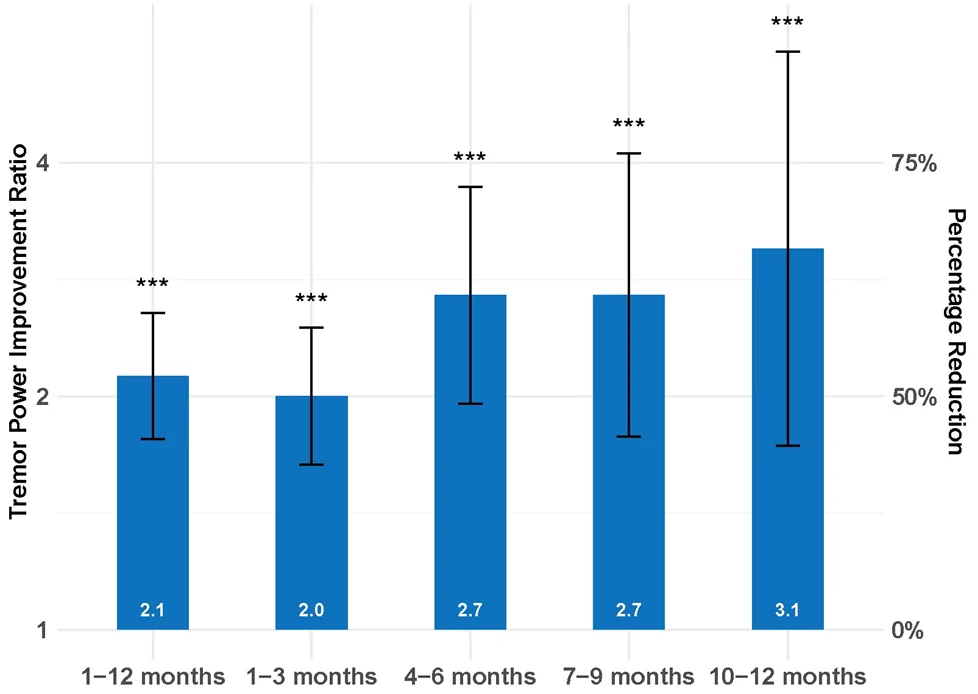
Tremor Power Improvement Ratio Over Different Follow-up Periods Over 12 Months

The prespecified tremor power endpoint was met. Over the 12-month follow-up period, TPIR was 2.1 ×/÷ 0.2 (*P* < .0001, **Figure 1**) and tremor power was reduced by 53.2%, from 0.079 ×/÷ 0.011 (m/s^2^)^2^ (geometric mean ×/÷ geometric standard error) pre-stimulation to 0.037 ×/÷ 0.005 post-stimulation. Across follow-up periods, TPIR ranged from 2.0 to 3.1 (**Figure 1**), corresponding to tremor power reductions of 50% to 67.7% (*P* < .0001). TPIR increased gradually across follow-up periods, reflecting a rise in pre-stimulation tremor power over time (**Supplementary Figure S4**). No significant differences in TPIR were found between the follow-up periods (*P* = .12).

The mean age was 67.7 years, with 72.4% aged 65 or older, 67.6% male, and 70% had received ET-related pharmacotherapy within 12 months prior to initiating TAPS therapy (including 32% with propranolol and 29% with primidone). On average, patients had 7.3 comorbid conditions.

**Figure 2.**
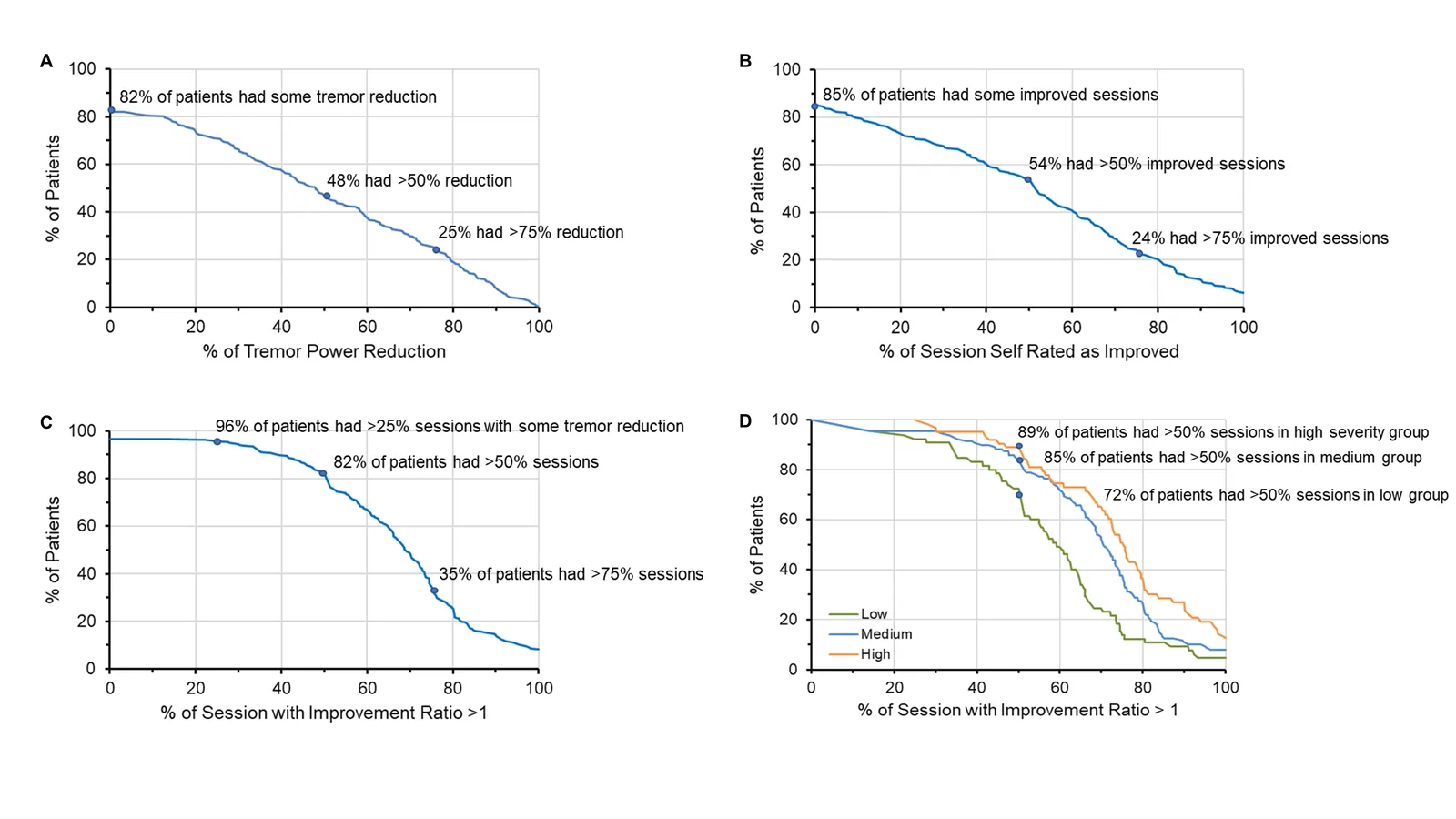
Objectively Measured and Subjectively Rated Tremor Improvement (**A**) Cumulative distribution of per-patient median tremor power reduction. (**B**) Cumulative distribution of per-patient percentage of sessions with self-rated tremor improvement. (**C**) Cumulative distribution of per-patient percentage of sessions with tremor power improvement ratio greater than 1. (**D**) Cumulative distribution of improvement ratio greater than 1 by tremor severity category.

Tremor power analysis showed that over 80% of patients had reduction (median TPIR >1) during typical sessions with nearly half experiencing greater than 50% reduction (median TPIR >2), and a quarter achieving greater than 75% reduction (median TPIR >4) (**Figure 2A**). Patient-reported ratings indicated most patients reported meaningful improvement in most of their sessions (**Figure 2B**).

To explore whether baseline tremor severity influenced therapy response, patients were stratified by median pre-stimulation tremor power across all sessions into low severity (0%-25% percentile), medium severity (25%-75% percentile), and high severity (75%-100% percentile) categories. Among these groups, over 70% of low severity and over 80% of medium and high severity patients experienced tremor reduction (TPIR >1) in more than half of their sessions (**Figure 2D**). Patients in the combined high and medium severity category experienced greater tremor improvement than patients in the low severity category (median TPIR: 4 vs 1; *P* < .0001). No significant differences in TPIR were observed by age groups (<65 vs ≥65 years) or sex.

Exploratory longitudinal analysis of the change in tremor power from pre-stimulation to pre-stimulation and from post-stimulation to post-stimulation were conducted. Pre-stimulation tremor increased significantly over time (*P* = .0023, mixed effects regression), while no significant post-stimulation tremor power changes were observed (*P* = .83).

**Figure 3.**
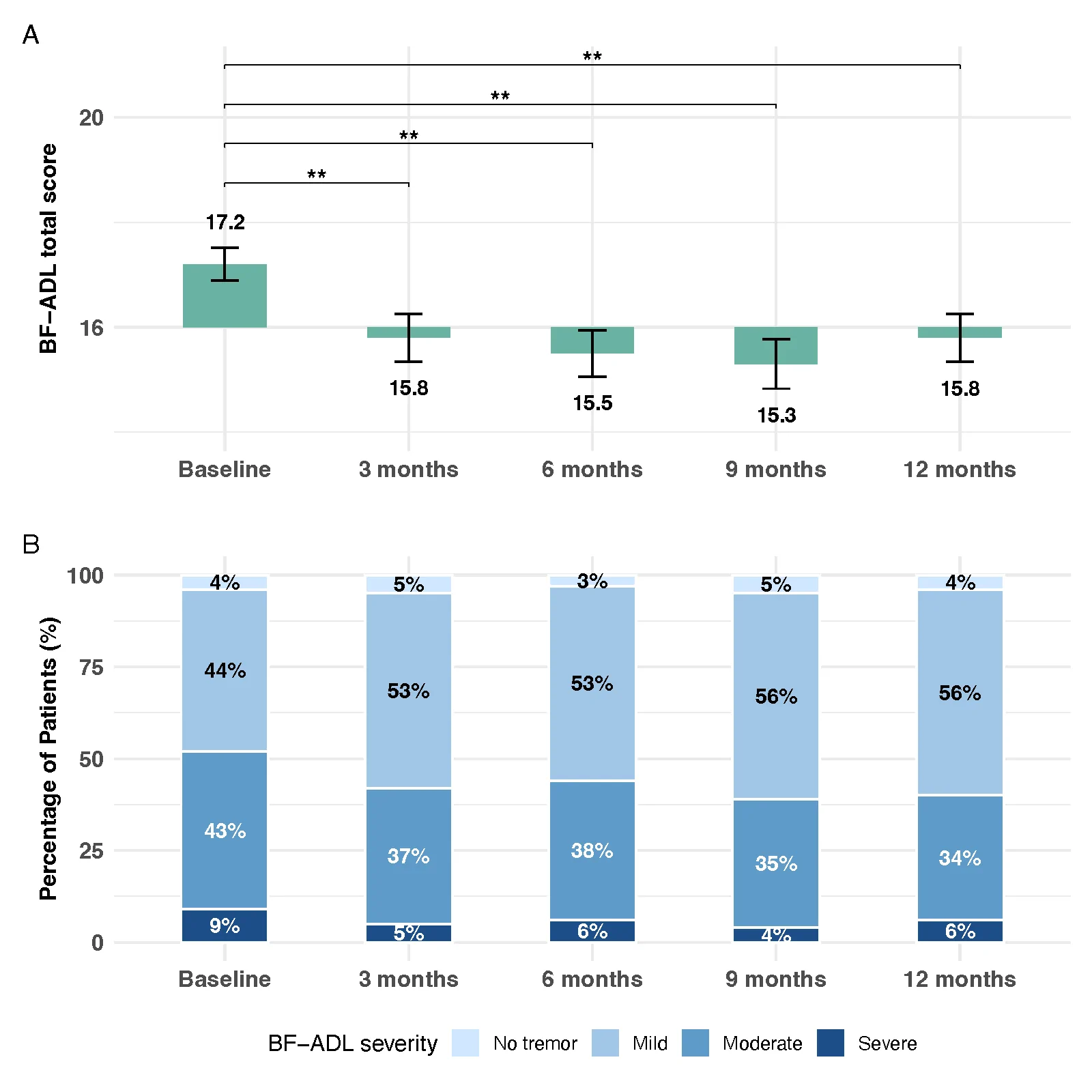
Bain and Findley Activities of Daily Living (BF-ADL) Scores (**A**) Total BF-ADL score at baseline and at different follow-up periods. Data are expressed as mean ± SD. (**B**) Tremor severity distribution (no tremor, mild, moderate, or severe) at baseline and follow-up periods. **Statistical significance (one sample *t*-test: *P* < .001).

The prespecified BF-ADL endpoint was also met, with significant reductions in BF-ADL scores from baseline to 12-month follow-up (1.4, *P* < .003, **Figure 3A**). Furthermore, significant improvements were observed at other follow-up timepoints (3-9 months: 1.4-1.9; *P* < .001) relative to baseline. No significant differences were found in BF-ADL scores between any follow-up timepoints from 3 months to 12 months (*P* = .59).

To assess the distribution of patients by BF-ADL severity across follow-up timepoints, patients were classified as follows: “No tremor” (total BF-ADL score: 8), “Mild” (9-16), “Moderate” (17-24), or “Severe” (25-32), which corresponds to average scores across the 8 assessed BF-ADL tasks: 1 (“able to do without difficulty”); between 1 and 2 (“able to do with little effort”); between 2 and 3 (“able to do with a lot of effort”); and between 3 and 4 (“cannot do without assistance”). Of patients with a BF-ADL score of “Moderate” or “Severe” at baseline, 34%, 24%, 36%, and 37% improved to “Mild” or “No tremor” at 3, 6, 9, and 12 months, respectively (all *P* < .0001) (**Figure 3B**). Compared with baseline, all 8 BF-ADL tasks significantly improved at some or all follow-up timepoints (*P* < .05, **Supplementary Table S3**).

### External Control Arm

Of the 225 patients who completed the 12-month TAPS therapy, 166 remained in the TAPS arm after excluding those without continuous enrollment and pharmacy benefits for the 12 months before and after the index date (**Supplementary Figure S2**). After applying all inclusion and exclusion criteria, 8106 eligible patients with ET were identified for the ECA (**Supplementary Figure S3**). Of the 166 TAPS patients with continuous enrollment and pharmacy benefits available, 153 patients (92.2%) were successfully matched to 2 non-TAPS patients. The matched cohort consisted of 459 patients (153 TAPS and 306 ECA), with a mean (SD) age of 67.5 (11.1) years; 32% were female; 69% were Medicare Advantage beneficiaries and 31% were covered by commercial health insurance. The mean (SD) CCI was 2.13 (2.22), and 71% had used at least 1 ET-related medication.

Before PSM, substantial differences existed between the TAPS arm and ECA, with TAPS patients generally younger and more likely to be male, White, commercially insured, and to have a higher comorbidity burden, greater ET-related medication use, and different socioeconomic characteristics (SMDs up to 0.84). After 1:2 PSM, 153 TAPS patients were matched to 306 ECA patients, achieving excellent balance across baseline characteristics (all SMDs <0.10, most <0.05). The matched cohorts were similar in demographics, comorbidity profiles, ET-related medication use, and baseline healthcare costs, including age (67.4 vs 67.6 years), male sex (68.0% vs 67.7%), all-cause costs ($13 640 vs $14 255), and ET-related costs ($1120 vs $1190) (**Supplementary Table S4**).

### HCRU and Costs

**Figure 4.**
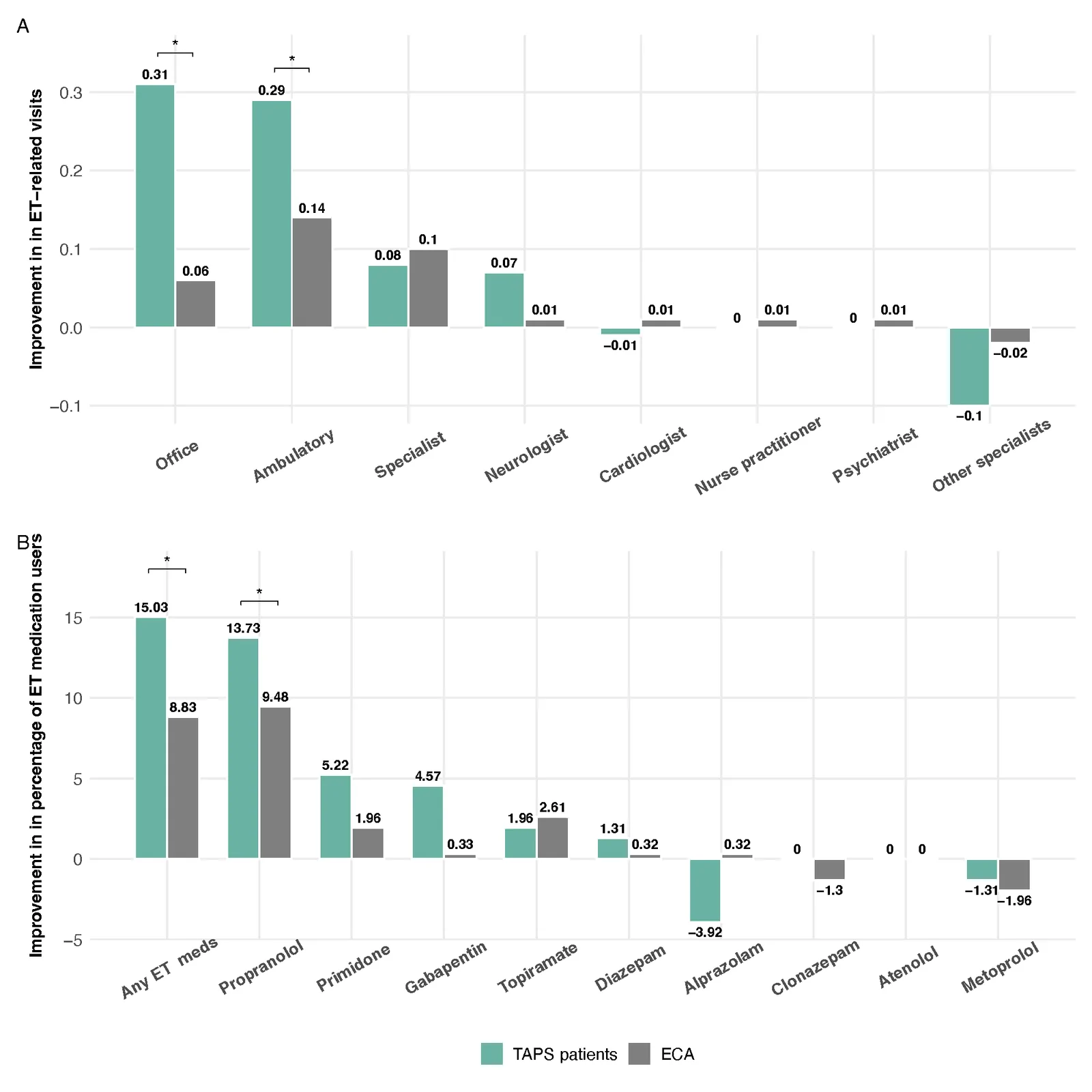
Change in Essential Tremor–Related (**A**) Healthcare Visits and (**B**) Medication Use from Baseline to 12-Month Follow-up in TAPS Patients and in ECA Abbreviations: ECA, external control arm; TAPS, transcutaneous afferent patterned stimulation. *Statistical significance (*P* < .05).

Matched TAPS patients showed a significant reduction in ET-related ambulatory (2.0 ± 1.8 to 1.7 ±1.3, *P* = .0238) and office visits (1.2 ± 1.3 to 0.9 ± 1.2, *P* = .0014) from baseline to follow-up, while non-TAPS patients in the ECA had no significant change (**Supplementary Table S5**). Furthermore, the TAPS patients demonstrated statistically significant reductions that were also significantly greater than those observed in the ECA for both ambulatory visits (0.3 ± 1.3 vs 0.1 ± 1.2, *P* = .0433) and office visits (0.3 ± 1.1 vs 0.1 ± 1.1, *P* = .0283) (**Figure 4A**). ET-related medication use decreased more in the matched TAPS patients (15% of patients) than in the ECA (8.8%, *P* = .0026), with a greater reduction in propranolol use (13.7% vs 9.5%, *P* = .0248, **Figure 4B, Supplementary Table S6**).

**Figure 5.**
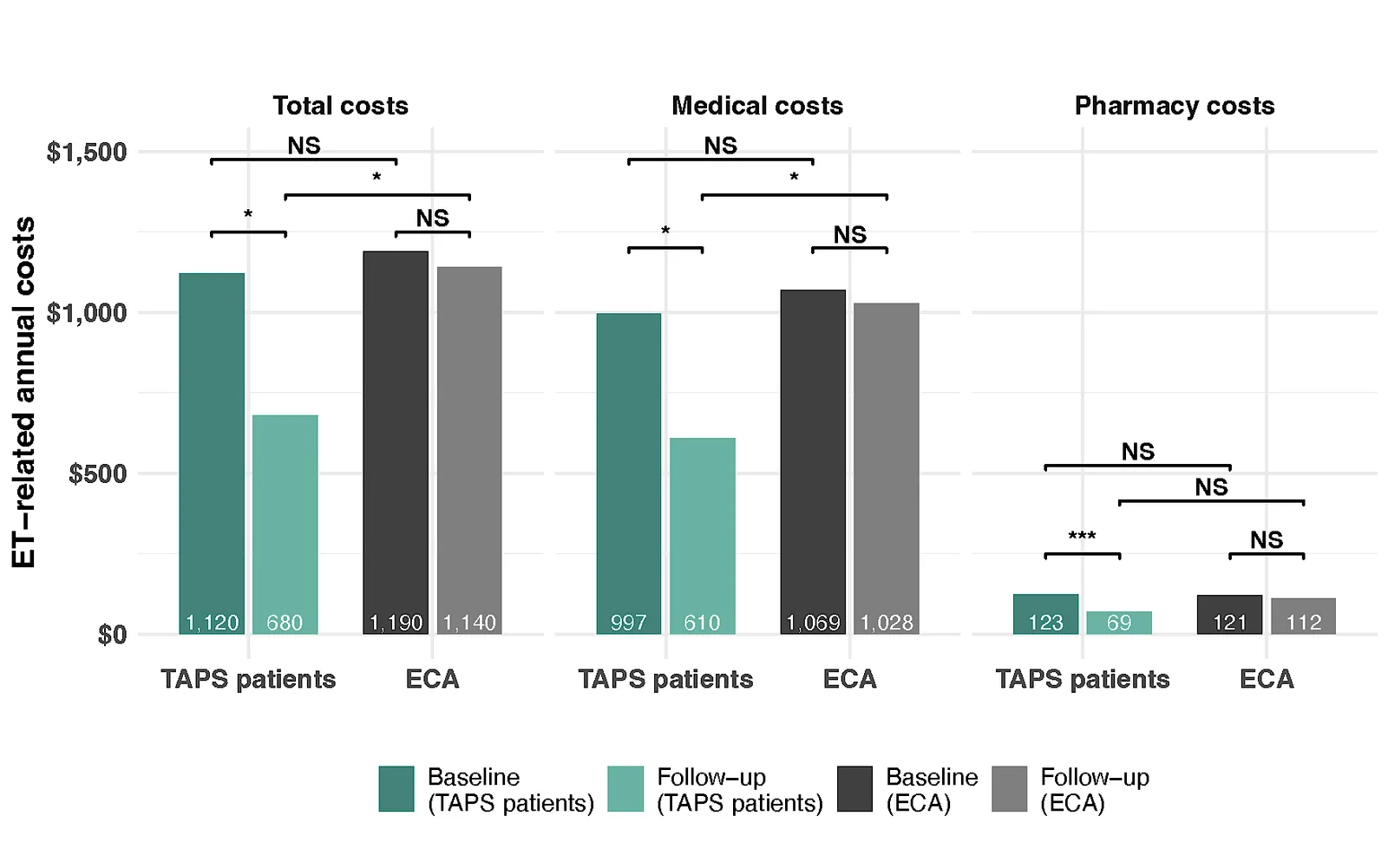
Comparison of Total ET-Related Healthcare Costs Between the Baseline and Follow-up Periods in TAPS Patients and the ECA Abbreviations: ECA, external control arm; NS, not statistically significant; TAPS, transcutaneous afferent patterned stimulation. Patients who used TAPS therapy over 12 months had significantly reduced ET-related healthcare costs compared with ECA. *P < .05. ***P < .0001.

Matched TAPS patients had a significantly greater reduction in ET-related costs compared to the ECA from the baseline to the follow-up ($440 ± 3498 vs $50 ± 5518, *P* < .0001), primarily driven by a decrease in medical costs ($387 ± 3471 vs $41 ± 5514, *P* < .0001, **Figure 5**). Pharmacy costs also declined more in the TAPS patients, though the difference was not statistically significant ($54 ± 174 vs $9 ± 157, *P* = .1501, **Figure 5**). Overall, TAPS therapy was associated with a significant decrease in total ET-related healthcare costs from baseline to follow-up ($1120 ± 3059 vs $680 ± 1763, *P* = .0023) and this decrease was greater than in the ECA.

No significant changes in all-cause HCRU or costs were observed in either arm. ET-related emergency department visits and inpatient admissions occurred too infrequently to analyze and are therefore not reported among the ET-related HCRU outcomes (**Supplementary Table S5**).

### Severe ET Subgroup Analysis

Most study patients (N = 187, 83%) met the criteria for the severe subgroup.^22^ TAPS therapy significantly reduced tremor power in this subgroup, with reductions ranging from 52.4% to 68.8% (TPIR of 2.1 to 3.2, *P* < .0001; **Supplementary Figure S5A**). No significant change in TPIR was observed between 1 and 12 months (*P* = .076, mixed effects regression). Analysis of the median tremor power improvement demonstrated that 85% of patients had some tremor reduction with 51% achieving more than a 50% reduction after their sessions.

BF-ADL significantly improved from baseline (1.6-2.2 points, *P* < .05, 1-sample *t*-test) across all follow-ups (**Supplementary Figure S5B**) with no significant BF-ADL changes observed from 3 to 12 months (*P* = .076, mixed effects regression). Patients with baseline scores ≥3 for a task showed significant improvement in that task at each follow-up (*P* < .01, **Supplementary Table S7**).

In the severe subgroup, ambulatory and office visits reduced by approximately 0.3 (*P* < .05, **Supplementary Figure S6A**), 17% fewer patients used ET-related medication (*P* = .0002, **Supplementary Figure S6B**), and average ET-related healthcare costs decreased by $442 (*P* = .008, **Supplementary Figure S6C**). ET-related HCRU and costs decreased significantly from the 12-month baseline to the 12-month follow-up period in matched TAPS patients ($1096-$654), with reductions significantly greater than those observed in the ECA group ($1197-$1024) (*P* < .01, **Supplementary Table S8**).

### TAPS Device Usage

TAPS patients administered therapy an average of 4.3 sessions per week and had at least 1 session for 3 days per week over 12 months. Additionally, patients in the severe ET subgroup used the TAPS therapy 4.4 sessions per week and applied at least 1 session for 3.1 days per week during the 12 months follow-up period (**Supplementary Table S9**).

### Adverse Events

Over 12 months, 41 device-related AEs were reported by 32 patients (10%, 32/310 enrolled patients). Most AEs were mild severity (78%, 32/41) with the remainder being moderate (22%, 9/41) (**Supplementary Table S10**). The most common reported AEs included wrist skin irritation (mild, N = 12; moderate, N = 1), tremor worsening (mild, N = 1; moderate, N = 4), minor electric shocking (mild, N = 4), uncomfortable sensation (mild, N = 4), and discomfort from stimulation (mild, N = 4). Of these AEs, 22% (9/41) occurred within the first month, while only 3 AEs were reported during months 10 to 12. All events were resolved without the need for professional medical attention. No severe AEs were reported. An additional 31 AEs were deemed unrelated to the device or therapy.

## DISCUSSION

This pragmatic clinical trial reinforces the long-term effectiveness and safety of TAPS therapy, demonstrating a reproducible acute reduction in tremor and improvement in daily function over 12 months. The therapy consistently reduced tremors during each session without evidence of tolerance over time, as post-stimulation tremor power remained stable and TPIR did not differ significantly across all follow-up periods. However, pre-stimulation tremor power increased over time, which is consistent with progression of the underlying condition. Therefore, the sustained benefits of this therapy should be interpreted as a reliable, reproducible reduction in symptoms rather than a halt of disease progression.^20,22–24^ The resulting gradual increase in TPIR across follow-up periods should not be interpreted as increasing efficacy; it reflects this rise in pre-stimulation tremor power over time, consistent with disease progression, while post-stimulation tremor power remained stable. By combining patient-reported outcomes, objective sensor data, and health claims, this study provides a comprehensive real-world assessment of TAPS therapy.

TAPS therapy significantly reduced tremor power and led to improvements in daily function, as measured by self-reported impact on ADL. While a minimal clinically important difference has not yet been established for the BF-ADL upper-limb scale, the observed improvements were statistically significant. These improvements were achieved by month 3 and maintained through month 12 with no significant variation across follow-up timepoints (*P* = .59), demonstrating an early and sustained functional benefit. Given that patients with ET consistently identify performing daily activities as one of their greatest challenges,^22,23^ these results highlight the clinical relevance of TAPS therapy. Together, these findings provide strong evidence that TAPS therapy delivers durable therapeutic and functional benefits in a real-world setting.

Overall, 10% of patients reported at least 1 AE, the majority of which were related to skin irritation or minor discomfort (eg, electronic shock, pain, burning, tingling) from stimulation. These AEs were typically mild and resolved without the need for professional medical attention. The proportion of patients who reported AEs, as well as the type and severity of these events, were consistent with findings from prior clinical trials and real-world evidence studies,^20,22–24^ further supporting the robust safety profile of TAPS therapy.

To our knowledge, this is the first real-world study to show a significant reduction in HCRU and costs among patients with ET who received TAPS therapy over 12 months compared to routine care. Patients using TAPS therapy experienced fewer ambulatory and office visits compared with baseline, along with a decrease in ET-related medication use, changes not observed in the ECA. Importantly, the patients with TAPS therapy demonstrated a significant reduction in overall ET-related healthcare costs, largely driven by lowered medical costs. These findings indicate that TAPS therapy was associated with reduced ET-related healthcare utilization and costs and with lower ET-related medication use, supporting its role as a noninvasive treatment option in the management of ET. To this point, a greater reduction in ET-related medication use was observed in the TAPS patients compared with the ECA cohort. Although some of these medications (eg, gabapentin, metoprolol) may also be used for other conditions, the PSM procedure accounted for relevant comorbidities and overall comorbidity burden (CCI). Thus, the decrease in medication utilization observed in the TAPS arm is most consistent with a tremor-specific therapeutic effect rather than changes in the management of other comorbid conditions. As expected, all-cause HCRU or costs did not differ between the TAPS patients and the ECA, likely reflecting the burden of comorbid conditions in the ET population, which can mask intervention-specific effects on broader healthcare outcomes.^6,7,12,34^ Over 12 months of use, TAPS therapy was associated with significant reductions in tremor severity and improvements in daily functioning, along with reductions in ET-related HCRU and costs. As a noninvasive therapeutic option, TAPS may represent an alternative treatment approach for adult patients with ET; however, direct comparisons with invasive interventions such as DBS or MRgFUS were beyond the scope of the current study.

On average, patients used the TAPS therapy 4.3 times per week, with older patients (≥65 years) using it more frequently than younger patients. The lower usage observed in this study compared to previous post-market surveillance data^22,23^ may reflect several factors. One explanation may be that this study took place during the COVID-19 pandemic, which may have influenced patterns of device use due to changes in healthcare access or daily routines. Another explanation may be that the prior real-world evidence analyses^22,23^ included patients who had completed a 90-day trial period with at least 10 sessions of 20 minutes or more, while the current study included patients who discontinued use prior to 90 days or used fewer than 10 times in their first 90 days.

Patients with more severe tremor demonstrated greater tremor reduction and improvement in ADLs compared with the overall population, consistent with previous studies.^20–24^ Reductions in ET-related healthcare costs were similar between the severe ET subgroup and the main cohort. This may be because patients in the severe subgroup, while perceiving their tremor as significant, likely had less severe disease compared to those who opted for surgical interventions and were excluded. Thus, the reduction in healthcare costs found in the severe patient cohort were comparable to those found in the main cohort. The consistent reduction in ET-related healthcare costs across evaluated subgroups (eg, age ≥65 years and prior ET medication use; see **Supplementary Subgroup Analysis, Table S11**) supports TAPS therapy as a broadly effective intervention.

This study has several strengths. First, it is the longest prospective study of TAPS therapy conducted to date, evaluating both clinical and health economic outcomes in real-world settings. Second, it employed an ECA to compare HCRU and costs between patients who received TAPS therapy and those who did not. Since all patients were selected from the same healthcare system, the ECA was constructed using the same eligibility criteria as the pragmatic TAPS clinical trial, ensuring comparability between the two arms. Additionally, the payor’s claims database provided a large and diverse sample of non-TAPS patients, enabling a well-powered analysis. Third, PSM was used to balance observed characteristics between the two arms, resulting in a well-matched cohort across key sociodemographic and clinical characteristics. Finally, the study’s broad inclusion criteria and integration of health claims data enhanced external validity and enabled a detailed evaluation of HCRU and costs.

Several limitations should be noted. First, like other administrative claims data, the Aetna claims database may contain coding inaccuracies, limitations or data entry errors that could lead to misclassification of diagnoses, procedures, or medication use.^4,35^

Second, although randomized controlled trials are the gold standard for establishing causal evidence, they are not always feasible or ethical. In such cases, ECAs derived from real-world data provide a practical alternative for contextualizing single-arm results.^36^ While PSM achieved well-matched cohorts, some residual confounding may remain. However, the lack of baseline differences in ET-related and all-cause healthcare costs suggests that any remaining confounding is likely minimal.

Third, the HCRU and costs analyses were limited to claims covered by insurance, excluding self-payments or out-of-pocket costs for medical care and medications.

Fourth, patients in the TAPS arm participated in a pragmatic clinical trial and may have received additional study-related follow-up and engagement compared with patients in the ECA. Although the study was designed to reflect real-world clinical practice and did not involve protocol-mandated physician visits or study-related medical care, participation in the trial itself may have influenced healthcare-seeking behavior and healthcare utilization patterns. Therefore, some caution is warranted when interpreting comparisons of HCRU and costs between the TAPS and ECA.

Fifth, the ECA lacked clinical outcome data, including tremor power and BF-ADL scores because these data were not captured in administrative claims databases.

Sixth, clinical outcome analyses were based on patients with available follow-up data. Although attrition and missing assessments may have introduced bias, no significant baseline differences were observed between patients who completed and those who discontinued the 12-month trial (all *P* > .05), suggesting limited attrition bias from measured characteristics. However, residual bias related to unmeasured factors or treatment response cannot be excluded, and findings should be interpreted accordingly.

Seventh, the BF-ADL instrument was the only patient-reported outcome measure (PROM) for ADLs, which was designed to evaluate the functional impact of tremor on ADLs commonly affected by ET and has been widely used in clinical trials.^20,23,28^ Although it is recommended by the Movement Disorder Society,^37^ and recognized by the Centers for Medicare & Medicaid Services for coverage decisions,^38^ it was not developed under current regulatory standards. Consequently, the use of BF-ADL may limit direct comparability with studies using more recently validated PROMs such as the TETRAS-PRO.^39–41^

Eighth, patients who underwent ET-related procedures such as DBS, MRgFUS, or botulinum toxin injections were excluded from both study cohorts. Because these therapies are often reserved for patients with more severe or treatment-refractory ET, this exclusion may have reduced representation of the most severely affected patients and truncated the severity distribution in both cohorts. This may have led to underestimation of overall HCRU and costs in both treatment arm and ECA. For example, no ET-related emergency visits or inpatient admissions were reported during the study, likely due to the exclusion of patients with prior ET-related surgeries.^42^ Exclusion of these patients from the study may limit the generalizability of the findings to patients with advanced ET.

Ninth, the economic analyses evaluated healthcare costs captured in administrative claims data and did not include the cost of TAPS therapy itself. Therefore, the observed reductions in ET-related healthcare costs should not be interpreted as net cost savings. Formal assessments of the overall economic value of TAPS therapy, including threshold, budget-impact, and cost-effectiveness analyses that incorporate device costs, are warranted and should be explored in future research.

Tenth, the study was conducted during the COVID-19 pandemic, a period characterized by substantial disruptions in HCRU, including reductions in elective procedures and changes in healthcare-seeking behavior. These factors may have influenced HCRU, potentially contributing to lower HCRU and healthcare cost estimates, particularly during follow-up. To reduce the potential impact of temporal differences between the TAPS arm and ECA, index year was included in the propensity score analyses, resulting in balanced enrollment periods between the two arms.

Eleventh, claims data do not capture ET duration or severity because ICD-10-CM codes lack this information. Consequently, these factors could not be included in the propensity score analyses, potentially resulting in residual confounding. However, the ECA used the same eligibility criteria as the TAPS trial, and propensity score models incorporated 52 covariates, helping to improve cohort comparability and reduce selection bias and confounding.

Lastly, the study population consisted of patients enrolled in a pragmatic clinical trial who received TAPS therapy and were linked to a large US commercial and Medicare Advantage claims database. Compared with the broader ET population, the study cohort may differ with respect to insurance coverage, healthcare-seeking behavior, socioeconomic status, and willingness to adopt novel device-based therapies. Therefore, the findings may not be fully generalizable to uninsured individuals, Medicaid populations, or patients receiving care in healthcare settings not represented in the study. However, the cohort included patients from diverse geographic regions across the US, and the demographic characteristics were generally consistent with those reported in previous ET studies,^4,23,24^ supporting the broader relevance of the findings.

## CONCLUSIONS

This is the first long-term, pragmatic clinical trial to evaluate TAPS therapy for hand tremors in adults with ET in a real-world, home-based setting. Over 12 months of use, the therapy significantly reduced tremor severity, improved daily functioning, and decreased HCRU and costs associated with ET. No serious device-related adverse events were reported, and the acute tremor-reducing effect was reproducible throughout the 12 months, with no attenuation of the per-session response. These findings reinforce results from previous clinical trials and real-world evidence studies, suggesting that TAPS therapy is a safe, durable, and effective treatment option for individuals with action tremor that interferes with daily activities.

### Disclosures

D.D. and J.F. are employees of CVS Healthspire^TM^ Life Sciences Solutions which received funding from Cala Health, Inc. to conduct this research project. C.L. and K.H.R. are employees of Cala Health, Inc.; the study was conducted during their employment. The authors report no other potential conflicts of interest.

### Meeting Presentation

Preliminary findings from this study were presented at the Movement Disorder Society (MDS) 2024 Annual Meeting and the American Academy of Neurology (AAN) 2025 Annual Meeting. The corresponding abstracts were subsequently published in *Movement Disorders* (2024;39:S728–S729) and *Neurology* (2025;104[7 suppl 1]:1549; doi:10.1212/WNL.0000000000208604).

### Registration

ClinicalTrials.gov identifier: NCT05540626.

## Supporting information

Online Supplementary Material

## Data Availability

The de-identified aggregate data underlying this study will be made available to qualified investigators upon reasonable request for the sole purpose of replicating the analyses presented herein. The HCRU and cost analyses were conducted using data from a large US healthcare claims database. The authors’ data-use agreement prohibits the sharing or redistribution of raw patient-level data to third parties.
