# Supplementary material for: Clinical and Economic Outcomes of Transcutaneous Afferent Patterned Stimulation for Essential Tremor
Source: J Health Econ Outcomes Res. 2026 Oct 22;13(2):191–202. doi: 10.36469/001c.170793 (PMC13695490; doi:10.36469/001c.170793)
Supplement: Supplementary file 1 [file jheor_2026_13_2_170793_372574.pdf]

Online Supplementary Material

Dai D, Lu C, Rosenbluth KH, Fernandes J. Clinical and Economic Outcomes of Transcutaneous Afferent Patterned Stimulation for Essential Tremor. *JHEOR.* 2026.13(2):191-202. 10.36469/jheor.2026.170793

Supplementary Method. Methods for Propensity Score Matching

Table S1. Reasons for Withdrawal from the 12-month TAPS Trial

Table S2. Baseline Sociodemographic and Clinical Characteristics of Patients Who Completed vs Discontinued the 12-Month Pragmatic Clinical Trial

Table S3. Average BF-ADL Individual Task and Overall Scores in Patients at Baseline and Follow-up Timepoints

Table S4. Baseline Sociodemographic and Clinical Characteristics among Patients with and without TAPS Therapy

Table S5. Healthcare Resource Utilization before and during TAPS Therapy, with Comparison to ECA

Table S6. Medication Use before and after TAPS Therapy, with Comparison to ECA

Table S7. Average Individual Task Improvement at Each follow-up in Patients with Bain and Findley Activities of Daily Living (BF-ADL) Score ≥3 at Baseline for Each Task

Table S8. Healthcare Costs before and after TAPS Therapy in Severe Subgroup, with Comparison to ECA

Table S9. TAPS Device Usage and Effectiveness by Severe Subgroup

Table S10. Adverse Events Associated with TAPS Therapy

Figure S1. External Control Arm Construction Process

Figure S2. Attrition Diagram for TAPS Arm

Figure S3. External Control Arm Patient Attrition Diagram

Figure S4. Tremor Power at Pre- and Post-Stimulation over Different Follow-up Periods over 12 Months

Figure S5. Tremor Power Improvement Ratio (TPIR) (A) and Total BF-ADL Score (B) over Different Follow-up Periods in the Severe ET Subgroup

Figure S6. Number of Essential Tremor-Related Healthcare Visits (A), ET-related Medication Utilization (B), and ET-Related Healthcare Costs (C) during 12-Month Baseline and during 12-Month TAPS Follow-up in the Severe Subgroup

Subgroup Analysis

Table S11. Device Usage and Effectiveness by Age Group

Table S12. Healthcare Costs before and after TAPS Therapy in Subgroups

This supplementary material has been provided by the authors to give readers additional information about their work.

Supplementary Methods. Methods for Propensity Score Matching

Propensity scores (PS) were estimated by multivariable logistic regression analyses to predict the probability of receiving transcutaneous afferent patterned stimulation (TAPS) therapy. The covariables were selected based on prior knowledge of the relationship between covariate and outcome.[1–5] These covariates included age, gender, race, geographic region, urban-rural, household income, social vulnerability index (SVI),[6] payer type, top 25 most common comorbid conditions in essential tremor (ET) population,[1,7,8] Charlson Comorbidity Index (CCI), ET-related medications, baseline all-cause and ET-related healthcare cost, and index year. There was a total of 52 covariables in the PS model. Propensity score matching (PSM) was then performed to select control patients who didn't receive TAPS therapy with characteristics similar to the patients who received TAPS therapy. This analysis used 2:1 nearest neighbor matching within PS calipers. Nearest neighbors matching within PS calipers was used to identify up to two control patients "closest" to each patient with TAPS therapy, within calipers of 0.2 standard deviations of the logit of the PS using the SAS macro GMATCH developed by Kosanke J and Bergstralh E.[9] Matching without replacement was used,[10] such that once a match was identified, that eligible non-TAPS patient could not be chosen again as a match. All matched patients were included in the final analysis cohort (Figure S2).

To check that the PSM approach effectively controlled for differences in characteristics between the two arms (TAPS patients vs. non-TAPS in ECA) in the cohort, we inspected the distribution of the PS between pre-matched patients with TAPS therapy and non-TAPS patients from the payor's claims database. In addition to graphic depiction of the distribution of the PS, descriptive statistics (mean ± standard deviation, median ± interquartile range, minimum, and maximum) were calculated. Descriptive statistics (mean± standard deviation, or frequency distributions) and standardized mean difference (SMD) were calculated to compare the distribution of covariates included in the PS model between the ECA-matched TAPS patients and the non-TAPS patients in ECA. To ensure accurate matching using PSM, a flexible approach was employed. If any covariate included in the PS model showed a SMD of 0.1 or greater, we revised the inclusion and coding of the covariate (e.g., converting from continuous to categorical, adding interaction or higher order terms) in the model, re-estimated the PS, and repeated the matching steps.[10] If there were remaining covariates that were not balanced after PSM, these unbalanced variables were included in multivariable analyses for double adjustment.[10,11]

Table S1. Reasons for Withdrawal from the 12-Month TAPS Trial

Reasons for withdrawal	Number of patients (%)
Lack of benefit	11 (4.0)
No longer wishes to participate	10 (3.6)
Difficulty using therapy	3 (1.1)
Discomfort following therapy	3 (1.1)
Does not fit into lifestyle	3 (1.1)
No longer on Aetna insurance	3 (1.1)
Tremor worsening	3 (1.1)
Diagnosis of Parkinson’s disease	2 (0.7)
Flare-up of existing medical condition	2 (0.7)
Allergic reaction	1 (0.4)
Bladder cancer and hip replacement	1 (0.4)
Brain-stem stroke	1 (0.4)
Developed dupuytren’s contracture	1 (0.4)
Diagnosed with cancer	1 (0.4)
Diagnosis of cervical spondylosis	1 (0.4)
Longer benefit desired	1 (0.4)
Pacemaker implant	1 (0.4)
Sacral stimulator implant	1 (0.4)
Unspecified	2 (0.7)

Abbreviations: TAPS, transcutaneous afferent patterned stimulation.

Table S2. Baseline Sociodemographic and Clinical Characteristics of Patients Who Completed vs Discontinued the 12-Month Pragmatic Clinical Trial

Characteristics	Total (N = 276)	Completed (N = 225)	Discontinued (N = 51)	*P* value
**Age (years),** Mean (SD)	68.21 (11.09)	67.68 (11.13)	70.56 (10.71)	0.0921
**Age Group (years),** N (%)				0.3644
22-44	11 (3.99)	9 (4.00)	2 (3.92)	
45-64	60 (21.74)	53 (23.56)	7 (13.73)	
65-74	124 (44.93)	101 (44.89)	23 (45.10)	
≥75	81 (29.35)	62 (27.56)	19 (37.25)	
**Gender,** N (%)				0.3556
Male	183 (66.30)	152 (67.56)	31 (60.78)	
Female	93 (33.70)	73 (32.44)	20 (39.22)	
**Race,** N (%)				0.3008
Black	5 (1.81)	5 (2.22)	0	
White	233 (84.42)	185 (82.22)	48 (94.12)	
Hispanic	3 (1.09)	3 (1.33)	0	
Asian	3 (1.09)	2 (0.89)	1 (1.96)	
More than one race	3 (1.09)	3 (1.33)	0	
Other	4 (1.45)	3 (1.33)	1 (1.96)	
Unknown	25 (9.06)	24 (10.67)	1 (1.96)	
**Payers,** N (%)				0.0021
Commercial insurance	88 (31.88)	81 (36.00)	7 (13.73)	
Medicare Advantage	188 (68.12)	144 (64.00)	44 (86.27)	
**Geographic region**, N (%)				0.4151
Midwest	78 (28.26)	61 (27.11)	17 (33.33)	
Northeast	60 (21.74)	50 (22.22)	10 (19.61)	
South	98 (35.51)	78 (34.67)	20 (39.22)	
West	40 (14.49)	36 (16.00)	4 (7.84)	
**Urban-rural**, N (%)				0.4641
Urban	60 (21.74)	50 (22.22)	10 (19.61)	
Suburban	83 (30.07)	64 (28.44)	19 (37.25)	
Rural	133 (48.19)	111 (49.33)	22 (43.14)	
**Household income**, $, Mean (SD)	65,461 (21,596)	65,990 (21,369)	64,105 (22,729)	0.5745
**SVI,** Mean (SD)	0.36 (0.18)	0.36 (0.18)	0.36 (0.18)	0.1668
**Top 25 most common comorbidities**				
Pain disorders	229 (82.97)	190 (84.44)	39 (76.47)	0.1714
Hyperlipidemia	225 (81.52)	184 (81.78)	41 (80.39)	0.8179
Hypertension	190 (68.84)	154 (68.44)	36 (70.59)	0.7651
Fatigue and sleep related disorders	124 (44.93)	99 (44.00)	25 (49.02)	0.5153
Osteoarthritis	115 (41.67)	93 (41.33)	22 (43.14)	0.8135
Obesity	112 (40.58)	94 (41.78)	18 (35.29)	0.3946
Anxiety	87 (31.52)	68 (30.22)	19 (37.25)	0.3291

Characteristics	Total (N = 276)	Completed (N = 225)	Discontinued (N = 51)	*P* value
Chronic pulmonary disease	77 (27.90)	66 (29.33)	11 (21.57)	0.2643
Diabetes mellitus	70 (25.36)	58 (25.78)	12 (23.53)	0.7390
Depression	69 (25.00)	51 (22.67)	18 (35.29)	0.0601
Diverticular disease	65 (23.55)	55 (24.44)	10 (19.61)	0.4624
Ischemic heart disease	65 (23.55)	51 (22.67)	14 (27.45)	0.6772
Peripheral vascular disease	57 (20.65)	47 (20.89)	10 (19.61)	0.8383
Chronic kidney disease	49 (17.75)	42 (18.67)	7 (13.73)	0.4044
Fall	49 (17.75)	40 (17.78)	9 (17.65)	0.9824
Cerebrovascular disease	42 (15.22)	33 (14.67)	9 (17.65)	0.5926
Cancer (malignant)	39 (14.13)	35 (15.56)	4 (7.84)	0.1534
Liver disease	30 (10.87)	28 (12.44)	2 (3.92)	0.0775
Kidney stone	29 (10.51)	23 (10.22)	6 (11.76)	0.7457
Osteoporosis	29 (10.51)	22 (9.78)	7 (13.73)	0.4065
Atrial fibrillation	28 (10.14)	26 (11.56)	2 (3.92)	0.1030
Substance use disorders	28 (10.14)	25 (11.11)	3 (3.88)	0.2641
Rheumatoid arthritis/ collagen vascular disease	23 (8.33)	19 (8.44)	4 (7.84)	0.8884
Iron deficiency anemia	21 (7.61)	18 (8.00)	3 (5.88)	0.6066
Stress and adjustment disorders	20 (7.25)	18 (8.00)	2 (3.92)	0.3104
**CCI,** Mean (SD)	2.23 (2.50)	2.12 (2.27)	1.77 (1.93)	0.2947
**Age-adjusted CCI,** Mean (SD)	4.78 (2.94)	4.81 (3.01)	4.65 (2.62)	0.7158
**Number of comorbidities,** Mean (SD)	7.20 (3.48)	7.31 (3.56)	6.75 (3.10)	0.2959
**Medication use,** N (%)				
**Medications for ET**				
Propranolol	86 (31.16)	72 (32.00)	14 (27.45)	0.5265
Primidone	82 (29.71)	66 (29.33)	16 (31.37)	0.7736
Topiramate	23 (8.33)	18 (8.00)	5 (9.80)	0.6739
Gabapentin	42 (15.22)	35 (15.56)	7 (13.73)	0.7425
Alprazolam	12 (4.35)	11 (4.89)	1 (1.96)	0.3545
Clonazepam	12 (4.35)	10 (4.44)	2 (3.92)	0.8687
Diazepam	4 (1.45)	3 (1.33)	1 (1.96)	0.7350
Metoprolol	40 (14.49)	34 (15.11)	6 (11.76)	0.5399
Atenolol	10 (3.62)	6 (2.67)	4 (7.84)	0.0741
Any ET related medications	190 (68.84)	157 (69.78)	33 (64.71)	0.4801
**Other medications**				
Antidepressants	77 (27.90)	59 (26.22)	18 (35.29)	0.1922
Anxiolytics	39 (14.13)	33 (14.67)	6 (11.76)	0.5911
Opioid use	50 (18.12)	41 (18.22)	9 (17.65)	0.9233
**Baseline BF-ADL severity**				
BF-ADL severity ≥2	262 (94.93)	214 (95.11)	48 (94.12)	0.7703
BF-ADL severity ≥3	224 (81.16)	186 (82.67)	38 (74.51)	0.1786

Characteristics	Total (N = 276)	Completed (N = 225)	Discontinued (N = 51)	*P* value
BF-ADL severity ≥4	75 (27.17)	63 (28.00)	12 (23.53)	0.5170
**Baseline BF-ADL score,** mean (SD)	17.14 (4.99)	16.98 (4.92)	17.89 (5.27)	0.2588
**Baseline tremor power**, $(m/s^2)^2$	0.056 (0.007)	0.057 (0.007)	0.051 (0.014)	0.7736

Data are expressed as mean (SD) for continuous variable, count (%) for categorical variables as indicated. Abbreviations: BF-ADL, Bain & Findley Activities of Daily Living; CCI, Charlson Comorbidity Index; ET, essential tremor; SD, Standard Deviation; SVI, Social vulnerability index; TAPS, transcutaneous afferent patterned stimulation; ECA, external control arm; $(m/s^2)^2$, geometric mean ×/÷ geometric standard error.

Table S3. Average BF-ADL Individual Task and Overall Scores in Patients at Baseline and Follow-up Timepoints

BF-ADL Task[1]	Baseline N = 225	3-month N = 203	6-month N = 181	9-month N = 160	12-month N = 148
Use a spoon to drink soup	2.4±0.8	2.2±0.8*	2.2±0.8**	2.2±0.7**	2.2±0.8
Hold a cup of tea	2.3±0.8	2.1±0.8*	2.1±0.8**	2.1±0.8*	2.2±0.8
Pour milk from a bottle/carton	2.0±0.8	1.9±0.8*	1.8±0.7**	1.9±0.8	2.0±0.8
Dial a telephone	1.9±0.8	1.7±0.8*	1.7±0.8*	1.6±0.8**	1.7±0.8
Pick up change	2.0±0.8	1.8±0.8*	1.9±0.8*	1.8±0.8**	1.9±0.8
Insert an electric plug	1.9±0.8	1.7±0.8**	1.7±0.8*	1.7±0.7*	1.7±0.7*
Unlock front door	1.9±0.7	1.7±0.8**	1.7±0.8**	1.7±0.7**	1.8±0.8
Write a letter	2.9±0.8	2.7±0.9**	2.8±0.9	2.7±0.9	2.8±1.0
Average per task	2.2±0.6	2.0±0.6**	2.0±0.6**	2.0±0.6**	2.0±0.6*

Data are expressed as mean±SD.

Abbreviation: BF-ADL, Bain and Findley Activities of Daily Living Scale.

[1] Each BF-ADL task was rated 1–4 by the patient (1 = able to do the activity without difficulty, 2 = able to do the activity with little effort, 3 = able to do the activity with a lot of effort, and 4 = cannot do the activity by yourself)

Multiple testing with Holm-Bonferroni correction was applied.

*$p < .05$ in comparison with baseline

**$p < .005$ in comparison with baseline

Table S4. Baseline Sociodemographic and Clinical Characteristics among Patients with and without TAPS Therapy

Characteristics	Before Propensity Score Matching			After Propensity Score Matching		
	**With TAPS** (N = 166)	**ECA** (N = 8,106)	**Standardized Difference**	**With TAPS** (N = 153)	**ECA** (N = 306)	**Standardized Difference**
**Age (years),** Mean (SD)	67.3 (10.8)	71.9 (10.3)	0.43	67.4 (10.9)	67.6 (11.2)	0.02
**Age Group (years),** N (%)						
22-44	6 (3.6)	209 (2.6)	0.06	6 (3.9)	8 (2.6)	0.07
45-64	37 (22.3)	972 (12.0)	0.28	33 (21.6)	73 (23.9)	0.06
65-74	84 (50.6)	3,545 (43.7)	0.14	78 (51.0)	147 (48.0)	0.06
≥75	39 (23.5)	3,380 (41.7)	0.40	36 (23.5)	78 (25.5)	0.05
**Gender,** N (%)						
Male	116 (69.9)	4,311 (53.2)	0.35	104 (68.0)	207 (67.7)	0.01
Female	50 (30.1)	3,795 (46.8)	0.35	49 (32.0)	99 (32.3)	0.01
**Race,** N (%)						
Black	3 (1.8)	212 (2.6)	0.06	3 (2.0)	6 (2.0)	0.00
White	137 (82.5)	3,669 (45.3)	0.84	125 (81.7)	253 (82.7)	0.03
Hispanic	2 (1.2)	56 (0.7)	0.05	2 (1.3)	3 (1.0)	0.03
Asian	1 (0.6)	37 (0.5)	0.02	1 (0.7)	2 (0.7)	0.00
More than one race	2 (1.2)	42 (0.5)	0.07	1 (0.7)	2 (0.7)	0.00
Other	2 (1.2)	91 (1.1)	0.01	2 (1.3)	5 (1.6)	0.03
Unknown	19 (11.5)	3,999 (49.3)	0.90	19 (12.4)	35 (11.4)	0.03
**Payers,** N (%)						
Commercial insurance	59 (35.5)	988 (12.2)	0.57	48 (31.4)	96 (31.4)	0.00
Medicare Advantage	107 (64.5)	7,118 (87.8)	0.57	105 (68.6)	210 (68.6)	0.00
**Geographic Region**, N (%)						
Midwest	48 (28.9)	2,362 (29.1)	0.01	47 (30.7)	84 (27.5)	0.07
Northeast	37 (22.3)	2,465 (30.4)	0.19	35 (22.9)	71 (23.2)	0.01
South	54 (32.5)	2,524 (31.1)	0.03	46 (30.1)	106 (34.6)	0.10
West	27 (16.3)	755 (9.3)	0.21	25 (16.3)	45 (14.7)	0.05
**Urban-rural**, N (%)						
Urban	39 (23.5)	1,444 (17.8)	0.14	37 (24.2)	62 (20.3)	0.09

Characteristics	Before Propensity Score Matching			After Propensity Score Matching		
	**With TAPS** (N = 166)	**ECA** (N = 8,106)	**Standardized Difference**	**With TAPS** (N = 153)	**ECA** (N = 306)	**Standardized Difference**
Suburban	43 (25.9)	2,100 (25.9)	0.03	40 (26.1)	87 (28.4)	0.05
Rural	84 (50.6)	4,562 (56.3)	0.11	76 (49.7)	157 (51.3)	0.03
**Household income**, $, Mean (SD)	65,479 (22,364)	60,944 (21,114)	0.21	65,616 (22,088)	64,803 (22,330)	0.01
**SVI,** Mean (SD)	0.35 (0.18)	0.41 (0.27)	0.23	0.36 (0.18)	0.36 (0.26)	0.02
**Top 25 most common comorbidities**						
Pain disorders	145 (87.4)	5,751 (71.0)	0.41	132 (86.3)	259 (84.6)	0.05
Hyperlipidemia	136 (81.9)	6,107 (75.3)	0.16	124 (81.1)	244 (79.7)	0.03
Hypertension	109 (65.7)	5,773 (71.2)	0.12	100 (65.4)	199 (65.0)	0.01
Fatigue and sleep related disorders	74 (44.6)	2,585 (31.9)	0.26	62 (44.5)	130 (42.5)	0.04
Osteoarthritis	70 (42.2)	2,856 (35.2)	0.14	63 (41.2)	129 (42.2)	0.02
Obesity	69 (41.6)	2,907 (35.9)	0.12	63 (41.2)	127 (41.5)	0.01
Anxiety	46 (27.7)	2,205 (27.2)	0.01	40 (26.1)	86 (28.1)	0.04
Chronic pulmonary disease	49 (29.5)	1,787 (22.1)	0.17	45 (29.4)	87 (28.4)	0.02
Diabetes mellitus	39 (23.5)	2,176 (26.8)	0.08	34 (22.2)	69 (22.6)	0.01
Depression	37 (22.3)	1,909 (23.6)	0.03	32 (20.9)	69 (22.6)	0.04
Diverticular disease	44 (26.5)	912 (11.3)	0.40	35 (22.9)	61 (19.9)	0.07
Ischemic heart disease	39 (23.5)	1,710 (21.1)	0.06	34 (22.2)	68 (22.2)	0.00
Peripheral vascular disease	37 (22.3)	1,556 (19.2)	0.08	32 (20.9)	62 (20.3)	0.02
Chronic kidney disease	29 (17.5)	1,270 (15.7)	0.05	27 (17.7)	63 (20.6)	0.08
Fall	29 (17.5)	961 (11.9)	0.16	26 (17.0)	52 (17.0)	0.00
Cerebrovascular disease	25 (15.1)	994 (12.3)	0.08	23 (15.0)	51 (16.7)	0.05
Cancer (malignant)	27 (16.3)	787 (9.7)	0.20	20 (13.1)	45 (14.7)	0.05
Liver disease	23 (13.9)	576 (7.1)	0.22	20 (13.1)	44 (14.4)	0.04
Kidney stone	18 (10.8)	388 (4.8)	0.23	16 (10.5)	31 (10.1)	0.01
Osteoporosis	16 (9.6)	960 (11.8)	0.07	13 (8.5)	27 (8.8)	0.01
Atrial fibrillation	22 (13.3)	732 (9.0)	0.14	20 (13.1)	45 (14.7)	0.05
Substance use disorders	19 (11.5)	1,100 (13.6)	0.06	18 (11.8)	31 (10.1)	0.05

Characteristics	Before Propensity Score Matching			After Propensity Score Matching		
	With TAPS (N = 166)	ECA (N = 8,106)	Standardized Difference	With TAPS (N = 153)	ECA (N = 306)	Standardized Difference
Rheumatoid arthritis/ collagen vascular disease	15 (9.0)	549 (6.8)	0.08	13 (8.5)	31 (10.1)	0.06
Iron deficiency anemia	14 (8.4)	394 (4.9)	0.14	11 (7.2)	27 (8.8)	0.06
Stress and adjustment disorders	15 (9.0)	434 (5.4)	0.14	13 (8.5)	22 (7.2)	0.05
**CCI,** Mean (SD)	2.2 (2.3)	1.8 (2.0)	0.16	2.1 (2.3)	2.2 (2.2)	0.03
**Number of comorbidities,** Mean (SD)	7.4 (2.6)	6.2 (3.3)	0.21	7.1 (3.4)	7.1 (3.4)	0.001
**Medication use,** N (%)						
**Medications for ET**						
Propranolol	53 (31.9)	1,379 (17.0)	0.35	49 (32.0)	100 (32.7)	0.01
Primidone	44 (26.5)	1,307 (16.1)	0.26	40 (26.1)	85 (27.8)	0.04
Topiramate	12 (7.2)	272 (3.4)	0.17	11 (7.2)	21 (6.9)	0.01
Gabapentin	28 (16.8)	788 (9.7)	0.21	26 (17.0)	50 (16.3)	0.08
Alprazolam	9 (5.4)	281 (3.5)	0.10	7 (4.6)	15 (4.9)	0.02
Clonazepam	4 (2.4)	268 (3.3)	0.05	4 (2.6)	7 (2.3)	0.02
Diazepam	2 (1.2)	108 (1.3)	0.01	2 (1.3)	5 (1.6)	0.03
Metoprolol	28 (16.9)	1,279 (15.8)	0.03	26 (17.0)	51 (16.7)	0.01
Atenolol	3 (1.8)	249 (3.1)	0.08	3 (2.0)	8 (2.6)	0.04
Any ET related medications	117 (70.5)	4,256 (52.5)	0.38	109 (71.2)	220 (71.9)	0.01
**Other Medications**						
Antidepressants	37 (22.3)	2,383 (29.4)	0.16	34 (22.2)	71 (23.2)	0.02
Anxiolytics	21 (12.7)	1,004 (12.4)	0.01	17 (11.1)	42 (13.7)	0.08
Opioid use	31 (18.7)	1,480 (18.3)	0.01	28 (18.3)	62 (20.3)	0.05
**All-cause healthcare costs ($)**						
Total medical	8,730 (13,240)	9,656 (19,917)	0.06	8,626 (13,556)	9,112 (11,899)	0.04
Total pharmacy	4,832 (14,218)	3,239 (12,010)	0.12	5,014 (14,753)	5,143 (15,190)	0.01
Overall	13,562 (20,836)	12,896 (24,509)	0.03	13,640 (21,535)	14,255 (23,064)	0.05
**ET-related healthcare costs ($)**						
Total medical	961 (2,943)	1,055 (4,851)	0.02	997 (3,040)	1,069 (3,566)	0.02

Characteristics	Before Propensity Score Matching			After Propensity Score Matching		
	**With TAPS** (N = 166)	**ECA** (N = 8,106)	**Standardized Difference**	**With TAPS** (N = 153)	**ECA** (N = 306)	**Standardized Difference**
Total pharmacy	127 (220)	77.31 (319)	0.02	123 (219)	121 (235)	0.02
Overall	1,088 (2,962)	1,132 (3,866)	0.01	1,120 (3,059)	1,190 (3,589)	0.02

Data are expressed as mean (SD) for continuous variable, count (%) for categorical variables as indicated. Abbreviations: CCI, Charlson Comorbidity Index; ET, essential tremor; SD, Standard Deviation; SVI, Social vulnerability index; TAPS, transcutaneous afferent patterned stimulation; ECA, external control arm.

Table S5. Healthcare Resource Utilization before and during TAPS Therapy, with Comparison to ECA

	TAPS Patients (N = 153)			ECA (N = 306)		
	**Baseline**	**Follow-up**	**Changes**	**Baseline**	**Follow-up**	**Changes**
**All-cause HCRU**, Mean (SD) unless otherwise specified						
Any ED visit, % (N)	20.3 (31)	14.4 (22)	-5.9	23.9 (73)	20.3 (62)	-3.6
Any inpatient admission, % (N)	7.8 (12)	5.9 (9)	-2.0	8.8 (27)	7.5 (23)	-1.3
Length of hospital stay (day)	0.3 (1.5)	0.3 (2.1)	-0.02	0.5 (2.2)	0.4 (1.9)	-0.1
Ambulatory visits	10.3 (7.3)	10.5 (7.5)	0.2	10.9 (7.3)	10.3 (7.6)	-0.6
Office visits	3.7 (2.6)	3.5 (2.9)	-0.2	4.8 (3.4)	4.5 (3.5)	-0.3
Specialist visits	6.6 (6.1)	6.9 (6.5)	0.3	6.8 (5.6)	6.7 (5.9)	-0.01
ED visits	0.3 (0.7)	0.2 (0.7)	-0.1	0.4 (0.8)	0.3 (0.7)	-0.1
Inpatient admissions	0.1 (0.3)	0.1 (0.3)	-0.01	0.1 (0.4)	0.1 (0.4)	0
Neurologist visits	0.5 (0.9)	0.4 (0.9)	-0.1	0.6 (1.0)	0.5 (0.9)	-0.2*
Cardiologist visits	0.4 (0.9)	0.5 (1.0)	0.1	0.5 (1.2)	0.5 (1.2)	0
Nurse practitioner visits	0.7 (1.4)	0.6 (1.0)	-0.1	0.7 (1.4)	0.5 (1.2)	-0.2*
Psychiatrist visits	0.2 (1.6)	0.3 (1.6)	0.1	0.1 (0.5)	0.1 (0.6)	0.01
Other specialists visit	4.8 (5.2)	5.2 (5.4)	0.4	4.6 (4.5)	4.6 (4.8)	0
**ET-related HCRU**, Mean (SD)						
Ambulatory visits	2.0 (1.8)	1.7 (1.8)	-0.3*	1.6 (1.4)	1.5 (1.8)	-0.1
Office visits	1.2 (1.3)	0.9 (1.2)	-0.3*	0.8 (1.1)	0.7 (1.3)	-0.1
Specialist visits	0.8 (1.2)	0.8 (1.4)	0.01	0.8 (1.1)	0.7 (1.2)	-0.1
Neurologist visits	0.4 (0.8)	0.3 (0.7)	-0.1	0.5 (0.9)	0.4 (0.9)	-0.1
Cardiologist visits	0.1 (0.3)	0.04 (0.3)	-0.01	0.03 (0.3)	0.02 (0.1)	-0.01
Nurse practitioner visits	0.1 (0.4)	0.1 (0.4)	0.01	0.1 (0.3)	0.1 (0.3)	0
Psychiatrist visit	0.01 (0.1)	0	-0.01	0.01 (0.1)	0.01 (0.2)	0
Other specialist visits	0.2 (0.6)	0.3 (0.8)	0.1*	0.2 (0.5)	0.2 (0.6)	0.02

McNemar’s test for binary variables. Paired-difference t-test and Wilcoxon signed-rank test for continuous variables.

*indicates $p < 0.05$ for comparison between baseline and follow-up within each arm. Abbreviations: TAPS, transcutaneous afferent patterned stimulation; ECA, external control arm.

Table S6. Medication Use before and after TAPS Therapy, with Comparison to ECA

	TAPS Patients (N = 153)			ECA (N = 306)		
	**Baseline**	**Follow-up**	**Changes**	**Baseline**	**Follow-up**	**Changes**
**ET-related medications**, % (N)						
Propranolol	32.0 (49)	18.3 (28)	-13.7**	32.7 (100)	23.2 (71)	-9.5**
Primidone	26.1 (40)	20.9 (32)	-5.2	27.8 (85)	25.8 (79)	-2.0
Topiramate	7.2 (11)	5.2 (8)	-2.0	6.9 (21)	4.3 (13)	-2.6*
Gabapentin	17.0 (26)	12.4 (19)	-4.6	16.3 (50)	16.0 (49)	-0.3
Alprazolam	4.6 (7)	8.5 (13)	3.9*	4.9 (15)	4.6 (14)	-0.3
Clonazepam	2.6 (4)	2.6 (4)	0	2.3 (7)	3.6 (11)	1.3*
Diazepam	1.3 (2)	0	-1.3	1.6 (5)	1.3 (4)	-0.3
Metoprolol	17.0 (26)	18.3 (28)	1.3	16.7 (51)	18.6 (57)	2.0
Atenolol	2.0 (3)	2.0 (3)	0	2.6 (8)	2.6 (8)	0
Any ET related medications	71.2 (109)	56.2 (86)	-15.0**	71.9 (220)	63.1 (193)	-8.8**
**Number of ET-related Medications**, Mean (SD)	1.1 (0.9)	0.9 (1.0)	-0.2	1.1 (0.9)	1.0 (1.0)	0.03
**Other medications**, % (N)						
Antidepressants	22.2 (34)	24.2 (37)	2.0	23.2 (71)	26.8 (82)	3.6
Anxiolytics	11.1 (17)	12.4 (19)	1.3	13.7 (42)	14.7 (45)	1.0
Opioids	18.3 (28)	24.8 (38)	6.5	20.3 (62)	22.9 (70)	2.6

McNemar's test for binary variables. Paired-difference t-test and Wilcoxon signed-rank test for continuous variables. **indicates $p < 0.001$, *indicates $p < 0.05$ for comparison between baseline and follow-up within each arm.

Abbreviations: TAPS, transcutaneous afferent patterned stimulation; ECA, external control arm.

Table S7. Average Individual Task Improvement at Each Follow-Up in Patients with Bain and Findley Activities of Daily Living (BF-ADL) Score ≥3 at Baseline for Each Task

BF-ADL Task	3-month		6-month		9-month		12-month	
	N	Improvement	N	Improvement	N	Improvement	N	Improvement
Use a spoon to drink soup	79	0.5 (0.1)**	71	0.6 (0.1)**	69	0.5 (0.1)**	65	0.5 (0.1)**
Hold a cup of tea	80	0.6 (0.1)**	72	0.6 (0.1)**	67	0.5 (0.1)**	66	0.5 (0.1)**
Pour milk from a bottle/carton	43	1.0 (0.1)**	40	0.9 (0.1)**	38	0.7 (0.1)**	34	0.9 (0.1)**
Dial a telephone	34	1.1 (0.1)**	37	0.9 (0.2)**	32	1.0 (0.2)**	30	0.9 (0.2)**
Pick up change	33	0.9 (0.1)**	35	0.5 (0.2)*	33	0.7 (0.2)**	27	0.8 (0.2)**
Insert an electric plug	37	1.1 (0.1)**	37	0.6 (0.1)**	32	0.7 (0.1)**	27	0.9 (0.2)**
Unlock front door	36	0.8 (0.1)**	33	0.6 (0.2)*	32	0.9 (0.1)**	28	0.8 (0.2)**
Write a letter	112	0.4 (0.1)**	103	0.2 (0.1)*	91	0.3 (0.1)*	91	0.3 (0.1)*

Data are expressed as mean (SE). ADL improvement presented as mean and SE to better reflect the precision of the estimate given large variation in responses. It was calculated as the BF-ADL score at the end of the follow-up period (3, 6, 9, or 12 months minus the BF-ADL score at baseline, multiplied by -1. A positive value indicates improved ADL from baseline over follow-up period.
**indicates $p < 0.0001$, *indicates $p < 0.01$.

Table S8. Healthcare Costs before and after TAPS Therapy in Severe Subgroup, with Comparison to ECA

	TAPS Patients			ECA		
	**Baseline**	**Follow-up**	**Changes**	**Baseline**	**Follow-up**	**Changes**
**Severe ET**		N = 127			N = 254	
ET-related costs ($)						
Total medical	960 (2,821)	577 (1,460)	-383 (3,096)	1,071 (3,842)	907 (3,736)	-164 (5,143)
Total pharmacy	136 (235)	77 (154)	-59 (188)**	126 (239)	117 (244)	-9 (162)
Overall	1,096 (2,848)	654 (1,501)	-442 (3,132)*	1,197 (3,865)	1,024 (3,746)	-173 (5,147)
All-cause costs ($)						
Total medical	8,758 (13,820)	10,296 (14,221)	1,538 (18,128)	9,074 (11,856)	10,204 (19,200)	1,130 (19,623)
Total pharmacy	5,467 (15,361)	5,290 (11,633)	-177 (6,077)	5,987 (20,567)	5,630 (18,758)	-357 (8,578)
Overall	14,225 (22,487)	15,587 (19,269)	1,362 (19,665)	15,061 (24,358)	15,834 (27,511)	773 (21,958)

Data are expressed as mean (SD).

Severe patients were classified as having at least one of 4 BF-ADL tasks which impacts eating, drinking, or writing score ≥3.[12]

**indicates $p < 0.0001$, *indicates $p < 0.01$ for comparison between baseline and follow-up within each arm.

Abbreviations: TAPS, transcutaneous afferent patterned stimulation; ECA, external control arm.

Table S9. TAPS Device Usage and Effectiveness by Severe Subgroup

	**All TAPS Patients** N = 225	**Severe** N = 187	**Less Severe** N = 38	***p* value**
**Usage patterns,** mean (SD)				
Sessions per week	4.3 (3.7)	4.4 (3.8)	4.1 (3.5)	0.08
Days per week with at least one session	3.0 (1.9)	3.1 (1.8)	2.8 (2.1)	0.06
Sessions per day on days when therapy used	1.3 (0.5)	1.3 (0.5)	1.2 (0.3)	0.19
**Tremor power**[1], geometric mean (SE)[2]				
Improvement ratio across all sessions	2.0 (0.2)	2.3 (0.2)	1.8 (0.4)	0.08
**BF-ADL rating**[3], mean (SD)				
Improvement	1.0 (4.3)	1.4 (3.9)	0.9 (4.2)	0.29
**Patient-rated outcomes,** % (N)				
Total sessions with patient-rating	10,238	8,521	1,717	
Improved	51.3 (5,247)	51.4 (4,376)	50.7 (871)	
No change	46.2 (4,729)	46.0 (3,916)	47.4 (813)	
Worsened	2.6 (262)	2.7 (229)	1.9 (33)	

[1]Device measured outcome; improvement ratio was defined pre-stimulation tremor power divided by post-stimulation tremor power.
[2]Geometric mean and standard error (SE) are analogous to arithmetic mean and SE of log-transformed data; and geometric SE represents x/÷ factor change from geometric mean.
[3]BF-ADL improvement, BF-ADL score changes defined as BF-ADL score at the end of 12 months minus BF-ADL score at baseline multiplied by -1. A positive value indicates improved BF-ADL from baseline over 12 months.
Severe patients were classified as having at least one of 4 BF-ADL tasks which impacts eating, drinking, or writing score ≥3.[12]
Abbreviations: TAPS, transcutaneous afferent patterned stimulation; ECA, external control arm; BF-ADL, Bain and Findley activities of daily living.

Table S10. Adverse Events Associated with TAPS Therapy

Event	Severity			Reported time			
	Mild	Moderate	Total	1-3M	4-6M	7-9M	10-12M
Persistent wrist skin irritation (redness, itchiness, rash, or swelling)	12	1	13	4	4	3	2
Tremor worsening	1	4	5	0	2	2	1
Minor shocking	4	0	4	2	2	0	0
Uncomfortable sensation	4	0	4	2	0	2	0
Discomfort from stimulation	3	0	3	2	0	1	0
Discomfort from stimulation and minor shocking	2	0	2	2	0	0	0
Minor skin irritation	2	0	2	0	1	1	0
Persistent discomfort from stimulation	3	0	3	0	1	2	0
Persistent discomfort from stimulation and minor shocking	1	0	1	1	0	0	0
Persistent discomfort from stimulation and persistent skin irritation	0	1	1	0	1	0	0
Persistent pain/discomfort from stimulation, and headache	0	1	1	1	0	0	0
Atrial fibrillation experience	0	1	1	0	1	0	0
Flare-up of fibromyalgia	0	1	1	1	0	0	0
Total	32	9	41	15	12	11	3

Abbreviation: TAPS, transcutaneous afferent patterned stimulation.

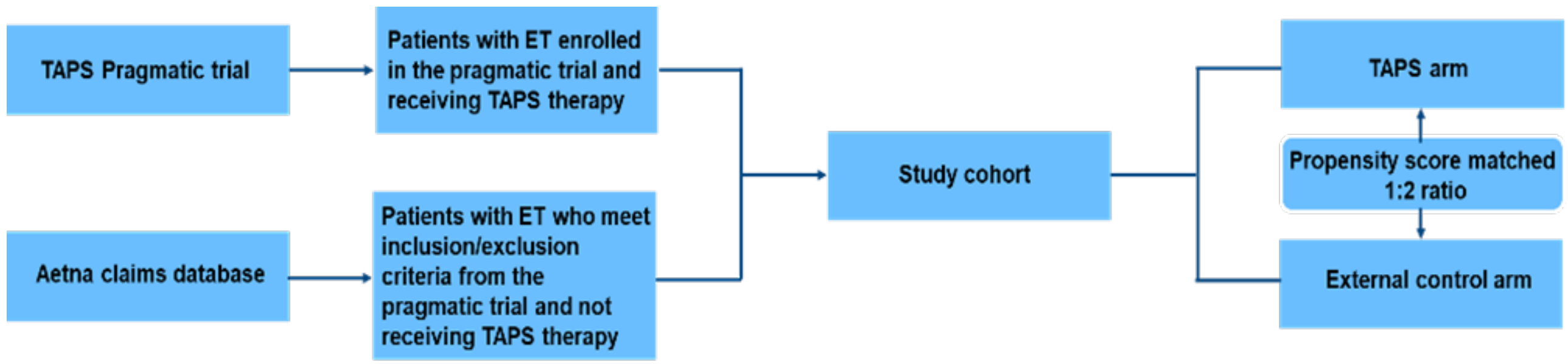

Figure S1. External Control Arm Construction Process

Abbreviations: ET, essential tremor; TAPS, transcutaneous afferent patterned stimulation.

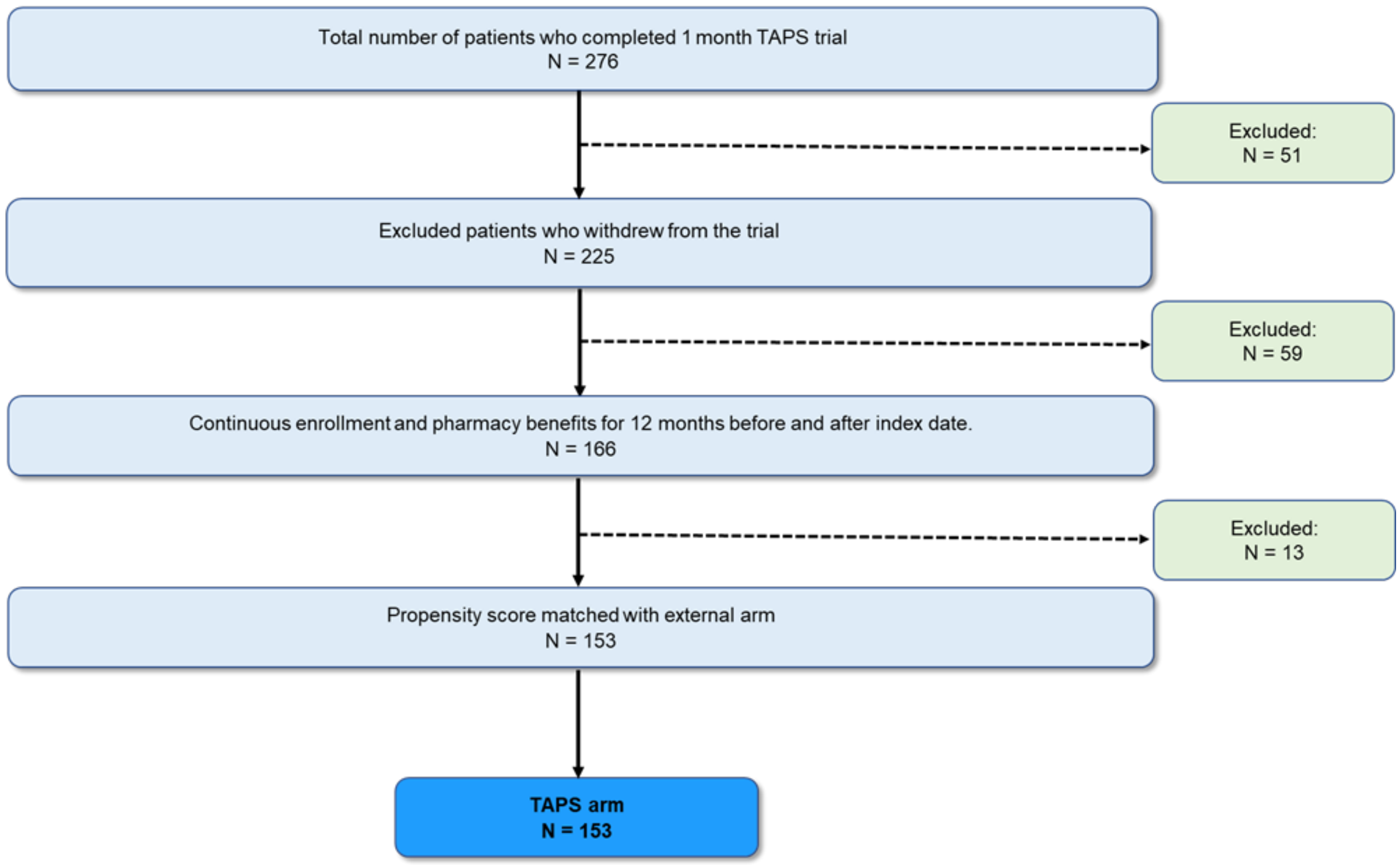

Figure S2. Attrition Diagram for TAPS Arm

Note: All 225 patients who used TAPS therapy at home were included in the analysis of clinical outcomes. Of these, 166 had continuous enrollment and pharmacy benefits available 12 months before and after the index date, from which 153 patients were successfully matched with the ECA using propensity scores were used to evaluate HCRU and costs.

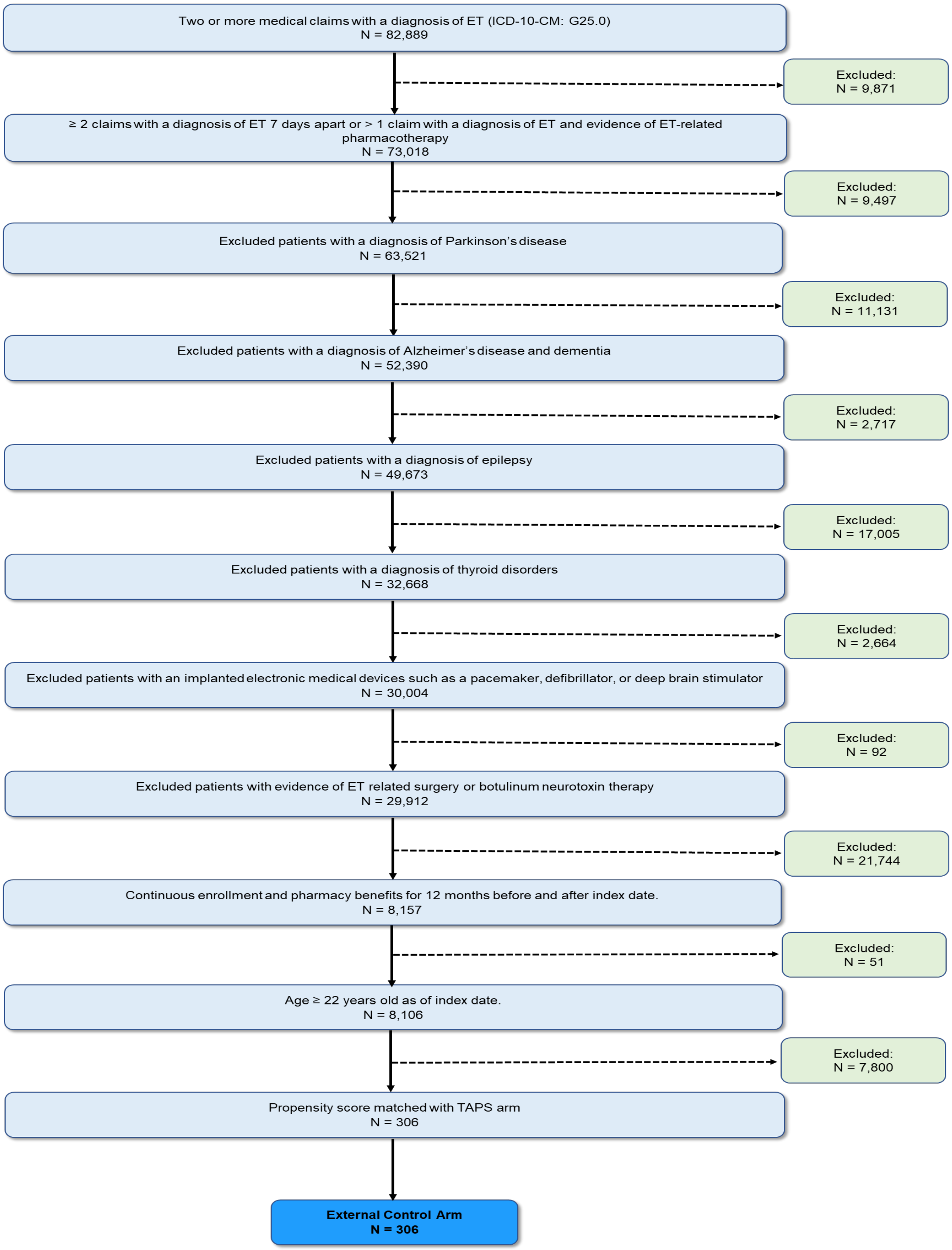

Figure S3. External Control Arm Patient Attrition Diagram

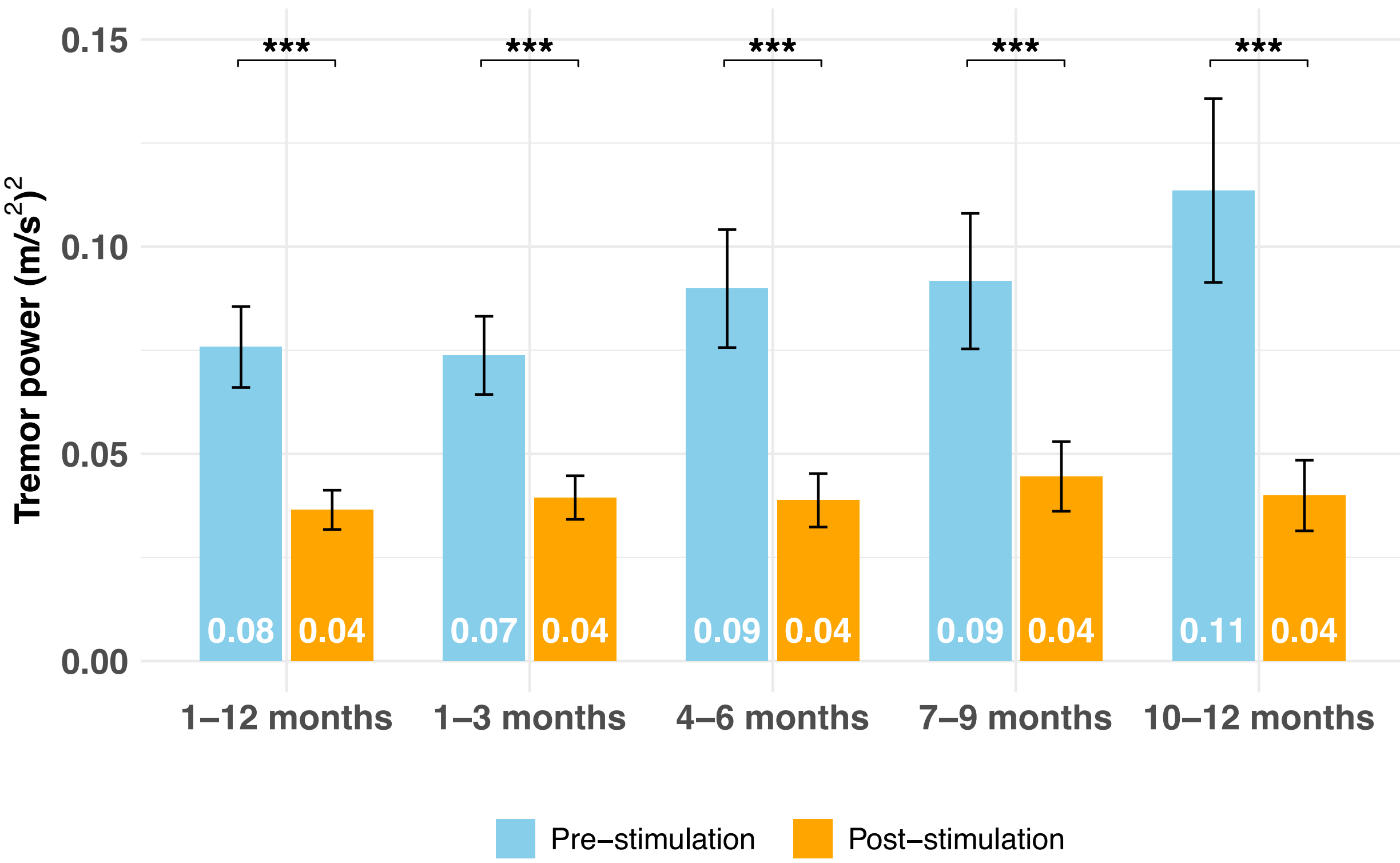

Figure S4. Tremor Power at Pre- and Post-stimulation Over Different Follow-up Periods Over 12 Months. Data is presented as geometric mean ± geometric standard error. TAPS demonstrated consistent efficacy in reducing tremor power in a real-world setting over 12 months. Asterisk (***) indicates statistical significance (p < 0.0001, Wilcoxon rank-sum test, pre- vs post-stim).

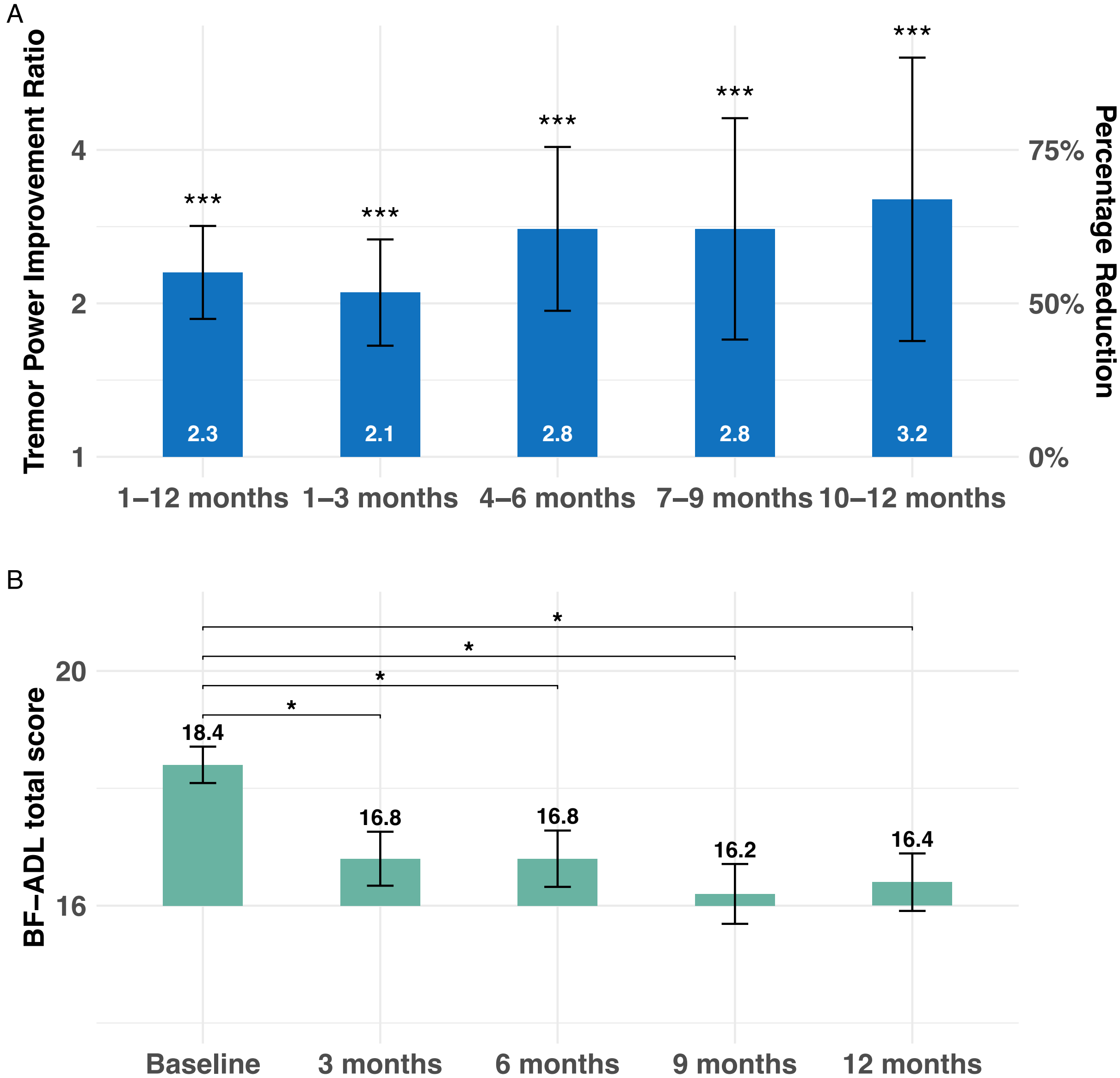

Figure S5. Tremor Power Improvement Ratio (TPIR) and Total BF-ADL Score Over Different Follow-up Periods in the Severe ET Subgroup (N=136). (**A**) TPIR over different follow-up periods in the severe ET subgroup. Data is presented as geometric mean and standard error of median tremor power improvement ratio. The Wilcoxon signed rank test to compare TPIR in the severe ET subgroup with the specified value of 1 during the specified follow-up period, all $p < 0.0001$. **(B)** Total BF-ADL Score at baseline and over different follow-up periods in the severe ET subgroup. Data is presented as mean and standard deviation. One sample t-test to compare BF-ADL

scores at the follow-up period vs baseline: *indicates $p < 0.05$; **indicates $p < 0.001$; ***indicates $p < 0.0001$.

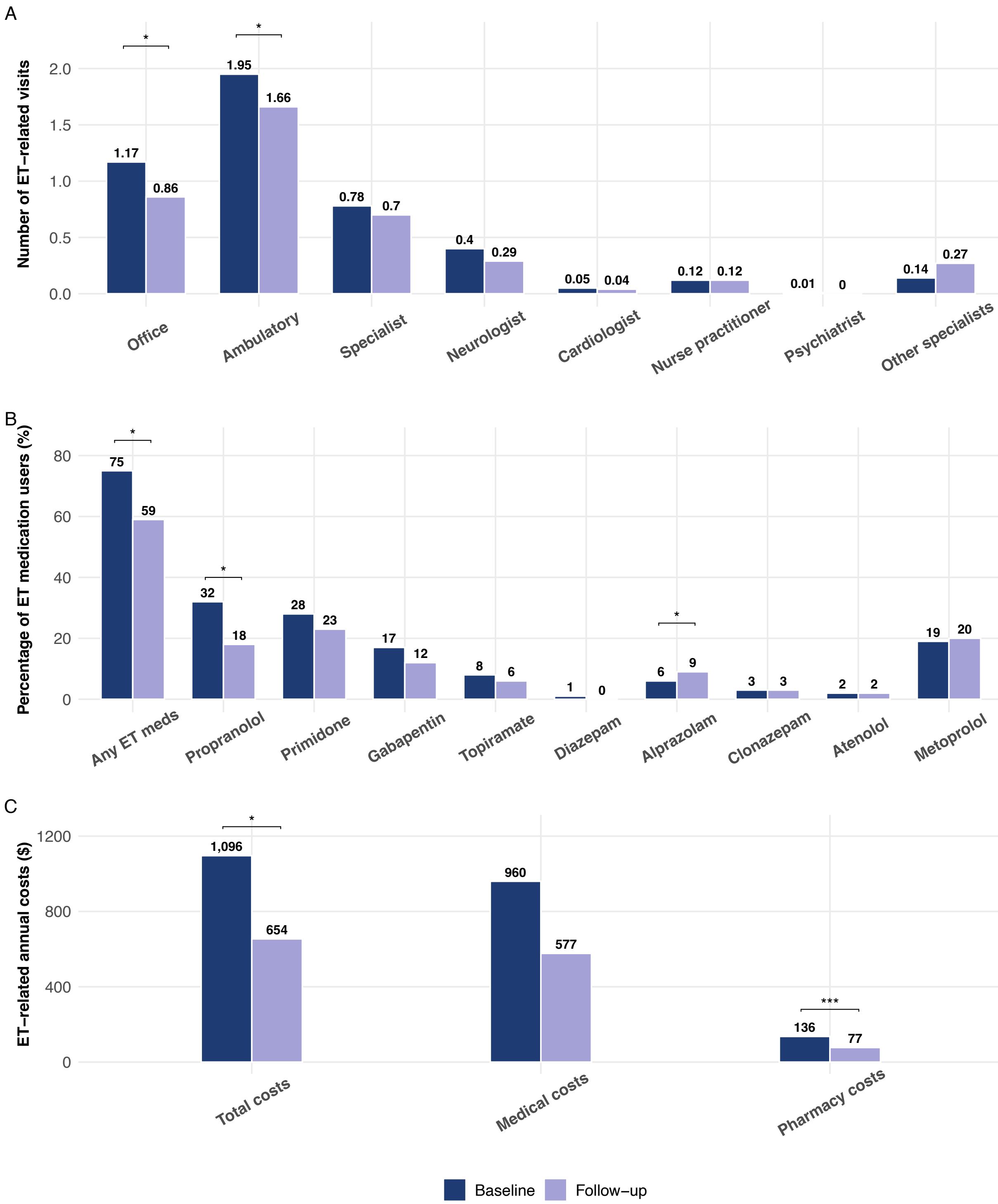

Figure S6. Comparison of Essential Tremor–Related Healthcare Visits (**A**), Medication Utilization (**B**), and Healthcare Costs (**C**) During the 12-Month Baseline and 12-Month

TAPS Follow-up Periods in the Severe Subgroup**.** *indicates p < 0.05; **indicates p < 0.001; ***indicates p < 0.0001.

Subgroup Analysis

Usage pattern and clinical outcome (exploratory analysis)

We investigated the effect of age on clinical outcomes in people with essential tremor by comparing patients aged ≥65 years to those aged <65 years. Patients aged ≥65 used the TAPS device more frequently than younger patients (4.6 vs. 3.4 sessions per week, $p = 0.045$; 3.3 vs. 2.3 days per week with at least one session, $p = 0.0006$; Supplementary Table 9). However, no significant differences were observed in tremor power reduction, functional outcomes, or patient ratings between age groups (Supplementary Table 9).

Healthcare resource utilization (HCRU) and costs (post hoc analysis)

We investigated the effect of older age and prior ET medication use on HCRU and costs in people with ET. Consistent with the main cohort, TAPS was associated with a significantly greater reduction in total ET-related healthcare costs. Notably, both older patients and those with prior ET medication use showed even greater cost reductions ($679 and $742, respectively) compared to the main cohort ($440). No significant changes in all-cause costs were observed in either group (Supplementary Table 10).

Table S11. Device Usage and Effectiveness by Age Group

	All Patients N = 225	Age < 65 N = 62	Age ≥ 65 N = 163	*p* value
**Usage patterns,** mean (SD)				
Sessions per week	4.3 (3.7)	3.4 (4.0)	4.6 (3.5)	0.045
Days per week with at least one session	3.0 (1.9)	2.3 (1.8)	3.3 (1.9)	0.0006
Sessions per day on days when therapy used	1.3 (0.5)	1.3 (0.5)	1.3 (0.5)	0.61
**Tremor power**[1], geometric mean (SE)[2]				
Improvement ratio across all sessions	2.0 (0.2)	2.6 (0.6)	1.9 (0.2)	0.07
**BF-ADL rating**[3], mean (SD)				
Improvement	1.0 (4.3)	1.6 (4.9)	0.9 (4.0)	0.40
**Patient-rated outcomes,** % (N)				
Total sessions with patient-rating	10,238	2,110	8,128	NA
Improved	51.3 (5,247)	49.4 (1,042)	51.7 (4,205)	NA
No change	46.2 (4,729)	48.2 (1,016)	45.7 (3,713)	NA
Worsened	2.6 (262)	2.5 (52)	2.6 (210)	NA

[1]Device measured outcome; improvement ratio was defined pre-stimulation tremor power divided by post-stimulation tremor power.

[2]Geometric mean and standard error (SE) are analogous to arithmetic mean and SE of log-transformed data; and geometric SE represents x/÷ factor change from geometric mean.

[3]BF-ADL improvement, BF-ADL score changes defined as BF-ADL score at the end of 12 months minus BF-ADL score at baseline multiplied by -1. A positive value indicates improved BF-ADL from baseline over 12 months.

Abbreviation: BF-ADL, Bain and Findley activities of daily living.

Table S12. Healthcare Costs before and after TAPS Therapy in Subgroups

	TAPS Patients			ECA		
	**Baseline**	**Follow-up**	**Changes**	**Baseline**	**Follow-up**	**Changes**
**Patients age ≥ 65 Years**		N = 105			N = 210	
ET-related costs ($)						
Total medical	1,255 (3,497)	642 (1,567)	-613 (3,787)*	1,312 (4,190)	1,347 (5,240)	35 (6,577)
Total pharmacy	129 (239)	64 (142)	-65 (189)**	117 (250)	108 (236)	-9 (176)
Overall	1,384 (3,519)	706 (1,614)	-678 (3,814)**	1,429 (4,217)	1,455 (5,243)	26 (6,581)
All-cause costs ($)						
Total medical	9,206 (11,244)	10,883 (19,293)	1,677 (18,882)	9,120 (11,025)	11,025 (21,012)	1,905 (21,487)
Total pharmacy	4,535 (15,395)	4,549 (10,975)	14 (6,501)	5,465 (20,646)	4,716 (17,269)	-749 (7,234)
Overall	13,741 (20,333)	15,433 (22,409)	1,691 (20,436)	14,585 (24,154)	15,741 (27,829)	1,156 (23,142)
**Used any ET medication at baseline**		N = 113			N = 226	
ET-related costs ($)						
Total medical	1,243 (3,500)	582 (1,526)	-661 (3,775)*	1,135 (631)	1,380 (5,470)	245 (6,274)**
Total pharmacy	184 (247)	103 (169)	-81 (197)**	200 (330)	161 (268)	-39 (227)*
Overall	1,427 (3,506)	685 (1,561)	-742 (3,799)**	1,335 (3,628)	1,541 (5,463)	206 (6,281)**
All-cause costs ($)						
Total medical	8,529 (10,666)	11,002 (19,095)	2,473 (18,792	9,397 (11,903)	11,597 (21,669)	2,200 (20,493)
Total pharmacy	6,163 (16,159)	6,127 (12,424)	-36 (6,794	6,001 (13,483)	5,791 (13,426)	-210 (6,303)
Overall	14,692 (21,590)	17,129 (22,845)	2,437 (18,792	15,398 (18,626)	17,388 (27,003)	1,990 (22,558)

Data are expressed as mean (SD).

**indicates $p < 0.0001$, *indicates $p < 0.01$ for comparison between baseline and follow-up within each arm.

Abbreviations: TAPS, transcutaneous afferent patterned stimulation; ECA, external control arm.

Supplementary References

1. Dai D and S Ali and Fernandes, Joaquim and Coetzer, Henriette. Multiple Comorbidities, Psychiatric Disorders, Healthcare Resource Utilization and Costs Among Adults with Essential Tremor: A Retrospective Observational Study in a Large US Commercially Insured and Medicare Advantage Population. J Health Econ and Outcomes Res. 2022;9(2):37-46. doi:10.36469/001c.37307

2. Kapinos KA, Louis ED. Annual health care costs among Medicare Beneficiaries with essential tremor. Parkinsonism Relat Disord. 2022;104:26-29. doi:10.1016/j.parkreldis.2022.09.015

3. Dai D, Fernandes J, Kim H, Coetzer H. Comparative Effectiveness of Transcutaneous Afferent Patterned Stimulation Therapy for Essential Tremor: A Randomized Pragmatic Clinical Trial. Tremor Other Hyperkinet Mov (N Y). 2023;13(1):38. doi:10.5334/tohm.798

4. Brookhart AM, Schneeweiss S, Rothman KJ, Glynn RJ, Avorn J, Sturmer T. Variable Selection for Propensity Score Models. Am J Epidemiol. 2006;163(12):1149-1156. doi:10.1093/aje/kwj149

5. Rassen JA, Glynn RJ, Brookhart AM, Schneeweiss S. Covariate Selection in High-Dimensional Propensity Score Analyses of Treatment Effects in Small Samples. Am J Epidemiol. 2011;173(12):1404-1413. doi:10.1093/aje/kwr001

6. Dai D, Fernandes J, Sun X, Lupton L, Payne VW, Berk A. Multimorbidity in atherosclerotic cardiovascular disease and its associations with adverse cardiovascular events and healthcare costs: a real-world evidence study. J Health Econ and Outcomes Res. 2024;11(1):75-85. doi:10.36469/ jheor.2024.94710.

7. Gerbasi ME, Nambiar S, Reed S, et al. Essential tremor patients experience significant burden beyond tremor: A systematic literature review. Front Neurol. 2022;13:891446. doi:10.3389/fneur.2022.891446

8. Okelberry T, Lyons KE, Pahwa R. Updates in essential tremor. Parkinsonism Relat Disord. March 2024:106086. doi:10.1016/j.parkreldis.2024.106086

9. Kosanke J, Bergstralh E. Gmatch SAS macro. 2024. Accessed July 6, 2024. https://bioinformaticstools.mayo.edu/downloads/sas/gmatch.sas.

10. Austin PC. Double propensity-score adjustment: A solution to design bias or bias due to incomplete matching. Stat Methods Med Res. 2016;26(1):201-222. doi:10.1177/0962280214543508

11. Austin PC. Balance diagnostics for comparing the distribution of baseline covariates between treatment groups in propensity-score matched samples. Stat Med. 2009;28(25):3083-3107. doi:10.1002/sim.3697

12. Brillman S, Colletta K, Borucki S, et al. Real-world evidence of transcutaneous afferent patterned stimulation for essential tremor. Tremor Other Hyperkinet Mov (N Y). 2022;12(1):27. doi:10.5334/tohm.715
